# Molecular phylogeny and bioprospecting of Endolichenic Fungi (ELF) inhabiting in the lichens collected from a mangrove ecosystem in Sri Lanka

**DOI:** 10.1371/journal.pone.0200711

**Published:** 2018-08-29

**Authors:** Kasun Maduranga, Renuka Nilmini Attanayake, Sinthujah Santhirasegaram, Gothamie Weerakoon, Priyani Ashoka Paranagama

**Affiliations:** 1 Department of Chemistry, University of Kelaniya, Kelaniya, Sri Lanka; 2 Department of Botany, University of Kelaniya, Kelaniya, Sri Lanka; 3 Integrative Research Centre, Department of Science & Education, Field Museum of Natural History, Chicago, United States of America; Mizoram University, INDIA

## Abstract

Endolichenic fungi (ELF) are unexplored group of organisms as a source for the production of bioactive secondary metabolites with radical scavenging activity, antilipase and amylase inhibitory activities. Endolichenic fungi in lichens collected from mangrove or mangrove associated plants are least known for their fungal diversity and potential to produce bioactive compounds. A total of 171 ELF strains were isolated from the lichens collected from mangrove and mangrove associated plants in Puttalam lagoon. Out of this collection, 70 isolates were identified using rDNA-ITS region sequence homology to the GenBank accessions and a phylogenetic analysis was performed. Commonly isolated genera of ELF from lichens were *Aspergillus*, *Byssochlamys*, *Talaromyces*, *Diaporthe*, *Phomopsis*, *Endomelanconiopsis*, *Schizophyllum*, *Cerrena*, *Trichoderma*, *Xylaria*, *Hypoxylon*, *Daldinia*, *Preussia*, *Sordaria*, *Neurospora*, and *Lasiodiplodia*. In the present study, the effectiveness of ethyl acetate extracts of the ELF isolates were investigated against antioxidant activity, antilipase activity and α-amylase inhibition activity in *in-vitro* conditions. The results revealed that the extracts of *Daldinia eschscholtzii*, *Diaporthe musigena and Sordaria* sp. had the highest radical scavenging activity with smaller IC_50_ values (25 μg/mL to 31 μg/mL) compared to the IC_50_ values of BHT (76.50±1.47 μg/mL). Antilipase assay revealed that 13 extracts from ELF showed promising antiobesity activity ranged between 25% to 40%. Amylase inhibitory assay indicated that the test extracts do not contain antidiabetic secondary metabolites.

## Introduction

Natural product research is moving forward impressively and interest in the exploration of microbial diversity has been encouraged by the fact that microbes serve as hidden treasures in discovering novel bioactive compounds. Interest in the discovery of new bioactive compounds from microbial sources have received greater attention than that of plants due to several facts such as easy maintenance of microbial cultures in liquid or solid states in controlled environments, limited space requirement and the requirement of small amount of initial inoculum safeguarding the biodiversity of the country [1]. However, it has been reported that less than 10% of the world’s biodiversity has been evaluated for potential biological activity although over 60% of the approved drugs were of natural origin [2]. Therefore, there is a great demand for secondary metabolites isolated from microorganisms with fascinating biological activities and most importantly, useful natural lead compounds with unique structural diversity can be discovered to aid drug discovery programs [3]. Among different types of microorganisms, fungi have gained a massive attention lately since fungi serve as metabolic factories and belong to the second largest kingdom of Eukaryots [3, 4]. Recent metagenomic studies suggested that there could be 3.5–5.1 million fungal species present on earth. However, only 5% of these species have been described by now [5] and only handful of species has been evaluated for their metabolic activities. In the present study it is assumed that some of these fungal species may be found in association with various ecological niches like lichens. Unlike other organisms, fungal species determination is challenging due to the availability of only a handful of few morphological characters such as pigmentation, conidia /spore length, width, mycelial colore and diameter, conidiophore diameter and length, dimensions of sexual structures [6]. Some of the morphological characters are plastic and can change accrodng to the environmental variations or nutritional conditions making it unreliable in species identification [6]. Compared to morphological characters, use of molecular characters, especially Internal Transcribed Spacer (ITS) region sequence data, has given promising results for species determination in various fungal species with the highest probability of success [5,7]. In a recent review, Raja et al. (2017) [6], highlighted the importance of the use of DNA barcode based species identification in fungal natural product chemistry. Therefore, DNA barcoding based species determination was used to describe endolichenic fungal species in Sri Lanka [6].

The endolichenic fungi (ELF) are the fungi that inhabit in lichen tissues asymptomatically without causing disease symptoms, and their occurrence is similar to the endophytic fungi live within healthy plant tissues [8]. It is evident that the ELF are important in producing chemically diverse secondary metabolites with novel anti-inflammatory, antioxidants and anticancer compounds [8, 9]. Dehydroherbarin, for example is isolated from an endolichenic fungal strain, *Corynespora* sp. occurring in the cavern beard lichen, *Usnea cavernosa* with significant inhibition of migration of human metastatic breast and prostate cancer cell lines, MDA-MB-231 and PC-3M, respectively [8]; two new biologically active compounds, PP-CT-01 and PP-CT-02, with anticancer and antioxidant activities were isolated from *Curvularia trifolii*, an endolichenic fungal strain in *Usnea* sp. in Sri Lanka, [9]; the isolation of two novel polyketides, PP-PC-01 and PP-PC-02 with radical scavenging activity in DPPH antioxidant assay were reported from *Penicillium citrinum*, an endolichenic fungal strain isolated from a *Parmotreama* sp. [10], showing the potential of the endolichenic fungi.

Mangrove ecosystems, located at the confluence of land and sea in subtropical and tropical coastal area show extraordinary adaptations as they are exposed to aggressive environmental conditions due to high salinity, low oxygen, strong winds and high light intensity [11]. Mangrove forests are distributed in tropical and sub-tropical countries and about 70 species of mangrove plants had been reported all over the world [11]. However, the literature review indicated that diversity and prevalence of endolichenic fungi in the lichens collected from mangrove ecosystems are untouched globally [12, 13]. U'Ren et al. (2012) found that endolichenic fungal diversity is affected by the climatic patterns, geographic separation, host type, and host lineage and therefore, studying endolichenic fungi in mangrove ecosystems would provide novel information for the scientific community [14]. The population of lichens on mangroves is different when compared to the lichens of terrestrial ecosystems and it may be due to the harsh environmental conditions prevailing in mangrove ecosystems [13] as their growth is hindered by the high level of salinity and moisture [12].

Being an island, Sri Lanka has several extensive coastal wetlands rich in mangrove forests covering approximately an area of 6000–7000 ha. The largest mangrove ecosystem in Sri Lanka is in Puttalam Lagoon covering 3385 ha [15]. Although Sri Lanka is a biodiversity hotspot, it is reported that prevalence and identification of lichens and microorganisms are understudied [15]. The mangrove forests in Sri Lanka and other countries have been damaged during past three decades with urban development, aquaculture and overexploitation [16]. Therefore, there is an urgent need of investigations aiming of isolation, identification and bioprospecting of mangrove-associated endolichenic fungi.

Considering the fact that harsh environmental conditions prevailing in mangrove ecosystems, it was hypothesized that ELFs of lichens living in mangrove ecosystems are rich in secondary metabolites. Since Sri Lankan mangrove associated ELF are untouched and no information is available, the present study was initiated. The main objectives were to identify lichen species and ELF isolated from the lichens of mangrove plants and mangrove associated plants, to report the fungal diversity, and to determine antioxidant, antilipase and amylase inhibition activities of secondary metabolites produced by solid cultures of ELF. This is the first comprehensive research on identification, phylogenic analysis and bioactivity of endolichenic fungi in mangrove ecosystem of Puttalam lagoon in Sri Lanka and this study would serve as a base line study for further studies on mangrove associated endolichenic fungi and isolation of bioactive natural products.

## Materials and methods

### Ethics statement

Field Permit: Forest Department of Sri Lanka granted permission to collect lichen samples from mangroves. The study sites were managed by the forest Department in Sri Lanka. The samples were collected from the trees that grew wildly in the area, where specific permit was obtained for taking samples. The trees used for sampling were treated ethically, and our study did not harm the local environment.

### Study site and sampling

**Fig 1:**
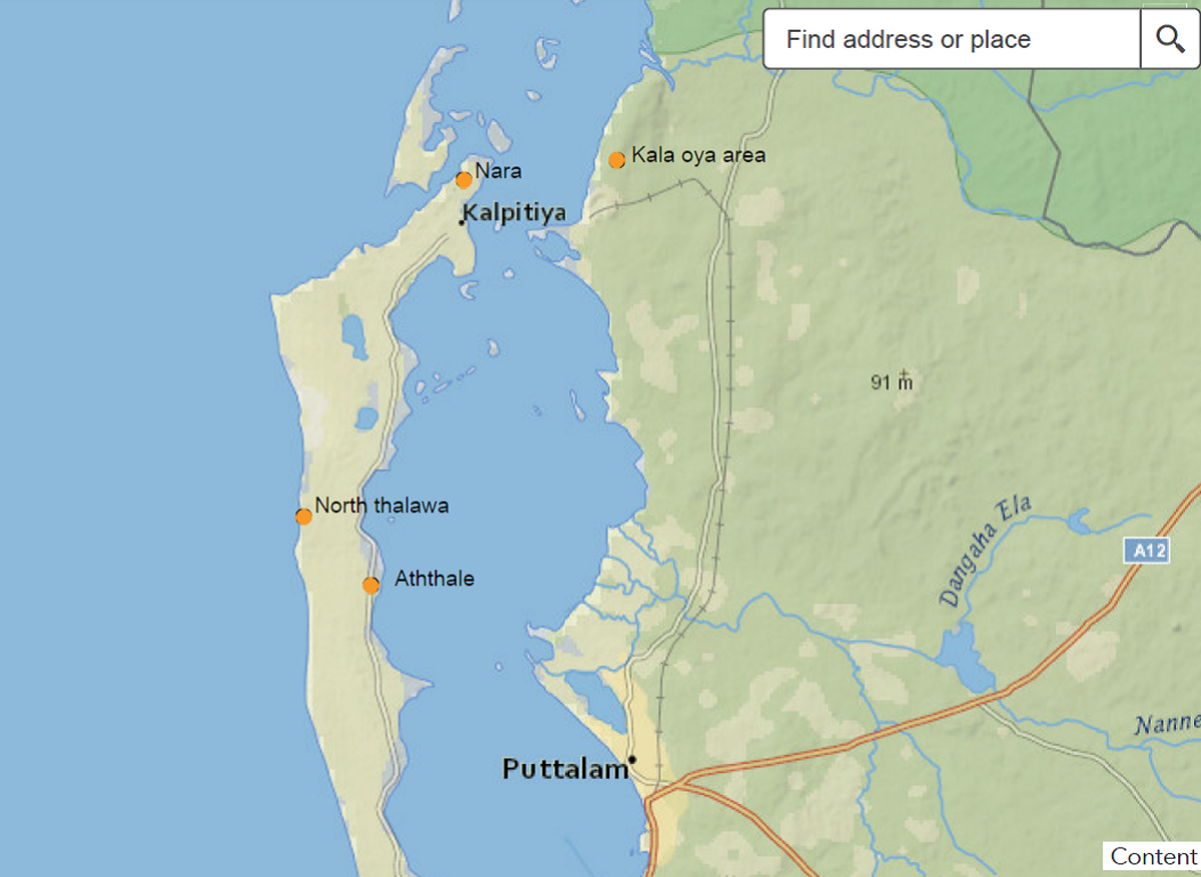
Sampling locations of lichens for the isolation of endolichenic fungi from mangrove study sites in Puttalam lagoon in Sri Lanka.

**Fig 2:**
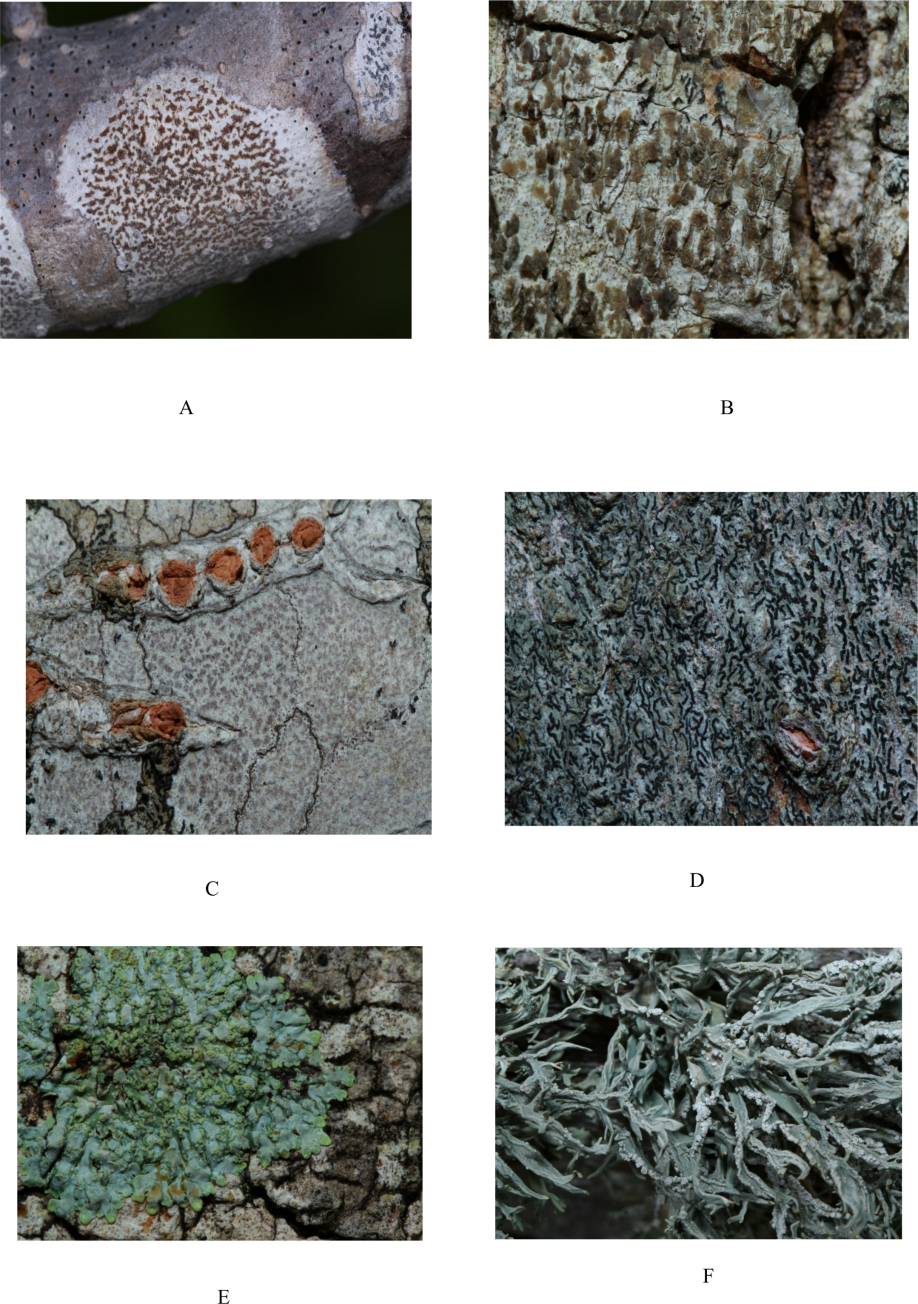
Selected lichens collected from mangrove and mangrove associated plants in Puttalam lagoon in Sri Lanka. (A) *Arthonia antillarum*. (B) *Arthonia antillarum*. (C) *Opegrapha medusulina*. (D) *Opegrapha medusulina*. (E) *Pyxine cocoes*. (F) *Rocella montagnei*.

The present study was based on an examination of 32 specimens of lichens collected during January 2016 to May 2016. The study was carried out in four sites; Aththale (8°05ˈ50.6ˈˈN and 79°43ˈ55.5ˈˈE), North–Thalawa (8°08ˈ17.3ˈˈN and 79°42ˈ22.6ˈˈE), National Institute of Aquatic Research Development Authority (NARA), (8°14ˈ53.9ˈˈN and 79°46ˈ17.3ˈˈE) and Kalaoya (8°17ˈ48.3ˈˈN and 79°50ˈ25.4ˈˈE) of Puttulam lagoon, Sri Lanka (Fig 1) and situated at an elevation of about 5 m above sea level. The lichens were collected from mangrove plants and mangrove associated plants of Puttalam lagoon. Lichens were collected from 18 mangrove species and 14 mangrove associated species. Samples were collected with stem bark / branch bark / areal part of host plants and immediately placed in acid free paper bags, labeled and transported to the Laboratory in the Department of Chemistry, University of Kelaniya. Samples were stored at 4°C and processed within 2 weeks. These lichens (Fig 2) were used to isolate endolichenic fungi.

### Lichen identification

The lichen samples (particle size 4×4cm) were kept in refridgerator for two weeks and air dried. Identification of the lichens was carried out at the Field Museum of Natural History, Chicago, U.S.A. and photographs were taken using Olympus stereomicroscopes and Olympus compound microscopes with interference contrast, connected to a Nikon Coolpix digital camera. All measurements were made on sections mounted in tap water. Voucher specimens were deposited in the Department of Chemistry, University of Kelaniya, Sri Lanka with a duplicate of each specimen [17].

### Isolation of endolichenic fungi

**Fig 3:**
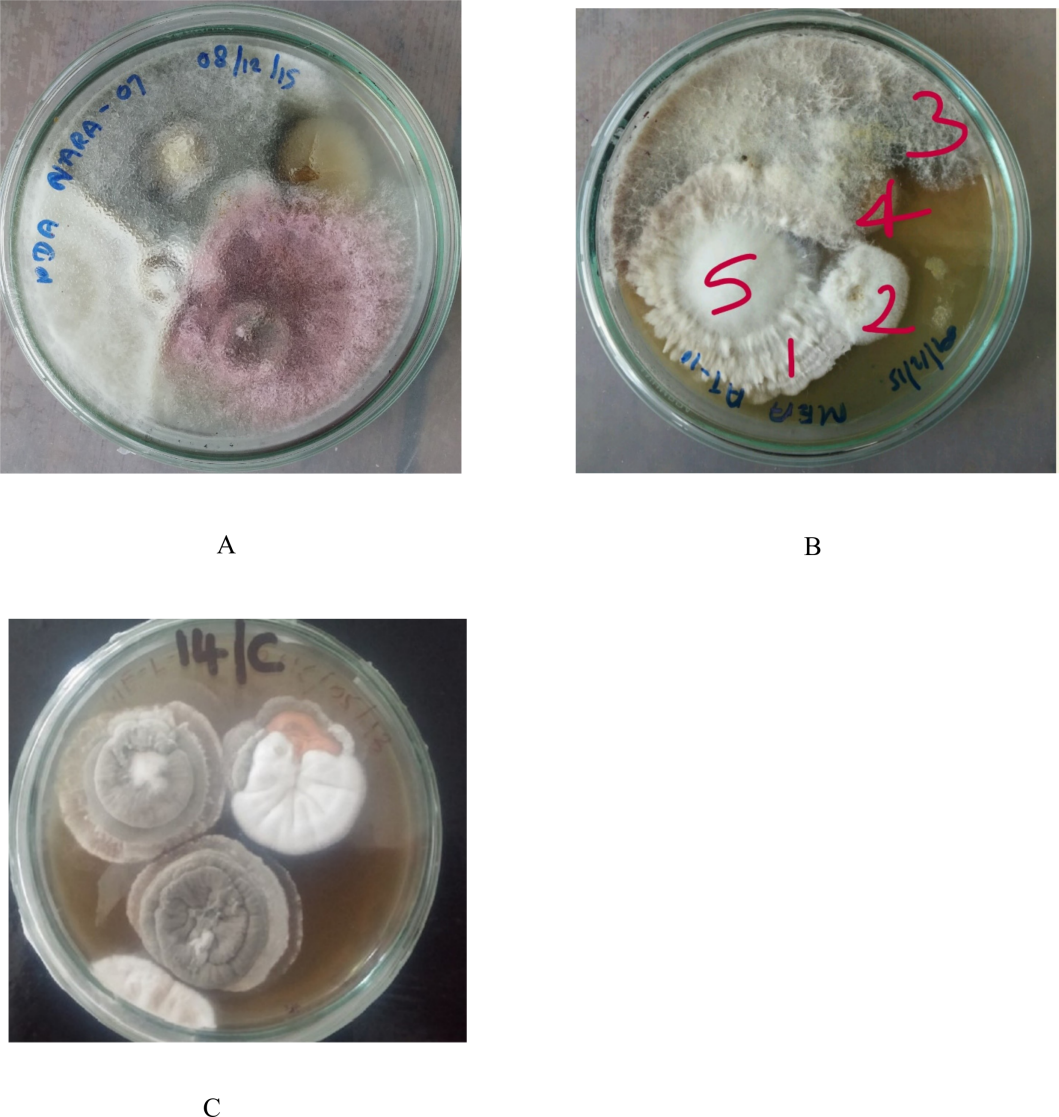
Endolichenic fungi emerging from the lichens. (A) Emergent endolichenic fungi from *Roccella montagnei*. (B) Emergent endolichenic fungi from *Opegrapha medusulina*. (C) Emergent endolichenic fungi from *Pyrenula indet*.

Healthy lichen thalli were cleaned with running tap water to eliminate suspended solids. Each lichen species was then cut into 1–2 cm^2^ segments and followed the protocol of Paranagama et al. (2007) [8] to remove surface microflora on lichen. The segments were dipped in 70% ethanol for 10 s, followed by 0.5% NaOCl for 3 min and then washed in sterilized distilled water for three times. The thalli were surface dried with sterile filter papers. After surface sterilization, five segments (approximately 3 x 3 mm) of each lichen species were placed on 2% malt extract agar (MEA) plate supplemented with 0.01% streptomycin. Plates were sealed with parafilm and incubated under ambient light/dark condition at room temperature (30°C) for 14 days. The tissue segments were observed periodically and fungi growing out of them were scored, isolated and sub-cultured (Fig 3). Finally, based on the morphological differences, the pure isolates were made and further incubated on PDA for one week [9]. Pure endolichenic fungal cultures were vouchered in sterile water, and deposited at the Department of Chemistry at the University of Kelaniya under strain ID as given in Table 1. Colony morphology on PDA plates and microscopic features, mycelial septation, pigmentation, branching pattern and sporulating structures, were observed under light microscopes. In total, 171 pure cultures of ELF strains were isolated from 32 lichen samples.

**Table 1:** Endolichenic fungal strains isolated from the lichens of mangrove and mangrove associated plants in Puttalam lagoon of Sri Lanka.

Name of the host plant	Lichen name	Strain ID	ELF from NCBI BLAST search	% Homology	GenBank Accession No.
*Aegiceras corniculatum*	Unidentified sterile sp.1	AT/L1/E6	*Schizophyllum commune* (KP689214)	99	MF773651
		AT/L1/E1ST	*Diaporthe arengae* (NR_111843)	99	MF773656
		AT/L1/E7	*Schizophyllum commune* (KP689214)	96	MF773657
*Soneratia* sp.	*Arthonia antillarum*	AT/L4/E3	*Lasiodiplodia theobromae* (KU977829)	100	KY992568
*Thespesia populnea* (Mangrove associated)	*Pyrenula parvinuclea*	AT/L5/E4	*Lasiodiplodia theobromae* (KP724987)	100	KY992574
*Thespesia populnea* (Mangrove associated)	Unidentified sterile sp. 2	AT/L3/E1	*Sordaria* sp. (KT823798.1)	100	KY992583
*Rhizophora neucronuta*	*Opegrapha medusulina*	AT/L6/E5	*Hypoxylon anthochroum* (KM516709.1)	100	KY992584
		AT/L6/E10	*Aspergillus hiratsukae* (KR909166)	100	KY977718
		AT/L6/E12	*Nigrospora sphaerica* (KX834821)	100	KY977719
		AT/L6/E1ST	*Diaporthe arengae* (NR111843)	99	MF773658
		AT-II/L6/E3	*Xylaria feejeensis* (JX256824)	100	MG593846
		AT-II/L6/E1	*Xylaria feejeensis* (JX256824)	100	KY992580
		AT-II/L6/E5	*Preussia* sp. (JN566152.1)	99	KY992581
*Cassia auriculata* (Mangrove associated)	*Pyrenula* parvinuclea	AT/L7/E1	*Neurospora crassa* (FJ360521)	100	KY992579
*Cocos nucifera(Mangrove associated)*	*cf*. *Roccella montagnei*	AT/L8/E1	*Neurospora* sp. (KT264374)	100	KY992575
		AT/L8/E1ST	*Aspergillus hiratsukae* (KR909166)	99	MF773659
		AT/L8/E5	*Daldinia eschscholtzii* (FJ624265)	100	KY977720
		AT/L8/E12	*Cerrena unicolor* (FN907915)	100	KY977721
*Cocos nucifera* (Mangrove associated)	*Opegrapha medusulina*	AT/L9/E1	*Daldinia eschscholtzii* (FJ624265)	100	MF773660
*Cocos nucifera*(Mangrove associated)	Unidentified sterile sp. 3	AT/L11/ElST	*Aspergillus fumigatus* (KP724987)	100	MF773652
		AT/L11/E1	*Endomelanconiopsis endophytica* (KF766164)	100	KY977723
		AT/L11/E3	*Aspergillus hiratsukae* (KR909166)	99	KY977724
*Cocos nucifera(Mangrove associated)*	*Pyxine cocoes*	AT/L12/E2	*Aspergillus hiratsukae* (KU761142)	99	MF773654
		AT/L12/E2ST	*Aspergillus hiratsukae* (KX960786)	99	KY9977725
		AT/L12/E4ST	*Neurospora crassa* (FJ360521)	100	KY992573
*Cocos nucifera* (Mangrove associated)	*Amandinea medusulina*	AT/L13/E2	*Xylaria psidii* (KU291350)	100	MF773655
*Excoecaria agallocha*	*Opegrapha arabica*	NT/L1/E1	*Daldinia eschscholtzii* (FJ624265)	100	KY992578
		NT/L1/E3	*Lasiodiplodia theobromae* (KP724987)	100	KY977731
*Excoecaria agallocha*	*Opegrapha medusulina*	NT/L2/E1	*Daldinia eschscholtzii* (FJ624265)	100	KY992576
*Excoecaria agallocha*	*Opegrapha medusulina*	NT/L3/E1	*Daldinia eschscholtzii* (FJ624265)	100	MF773669
		NT/L3/E2	*Daldinia eschscholtzii* (FJ624265)	100	KY992577
*Salvadora persica*	*Arthonia antillarum*	N/L1/E3	Xylariaceae sp. (AB440128)	100	MF773661
*Excoecaria agallocha*	*cf*. *Roccella montagnei*	N/L2/E4	*Daldinia eschscholtzii* (FJ624265)	99	MF773663
		N/L2/E7	*Xylaria castorea* (JF908802)	99	MF773662
*Syzygium* sp.	*Arthonia antillarum*	N/L4/E11	*Diaporthe musigena* (JF951138)	100	KY977726
		N/L4/E23	*Diaporthe arengae* (NR111843)	99	MF773664
		N/L4/E23ST	*Diaporthe arengae* (MF773656)	99	KY977727
*Syzygium* sp.	*Pyxine cocoes*	N/L5/E2	*Daldinia* sp. (KC343208)	100	MF773665
*Cocos nucifera (Mangrove associated)*	*Arthonia parantillarum*	N/L6/E1	*Preussia tenerifae* (EU551191.1)	100	KY992582
*Cassia auriculata* (Mangrove associated)	*cf*. *Roccella montagnei*	N/L7/E3	*Nigrospora* sp. (KX650827.)	100	KY992566
		N/L7/E6	*Rigidoporus vinctus* (KX549789.)	100	KY992567
*Cassia auriculata* (Mangrove associated)	*cf*. *Roccella montagnei*	N/L8/E2	*Lasiodiplodia theobromae* (KP724987)	100	KY99257
		N/L8/E1	*Lasiodiplodia pseudotheobromae* (LC270866)	99	KY977728
*Cocos nucifera(Mangrove associated)*	*cf*. *Roccella montagnei*	N_L9_E1	*Lasiodiplodia theobromae* (KP724987)	100	KY992570
		N/L9/E4	*Daldinia eschscholtzii* (FJ624265)	100	KY977729
*Cassia auriculata* (Mangrove associated)	*Pyrenula ochraceoflava*	N/L10/E10ST	*Lasiodiplodia theobromae* (KU977829)	100	KY992569
		N/L10/E4ST	*Byssochlamys spectabilis* (KP724987)	100	KY977730
*Thesphesia populnea* *Thesphesia populnea*	*Porina tetracerae* *Porina tetracerae*	KO2_23	*Daldinia eschscholtzii* (FJ624265)	100	MF773682
		KO2_7	*Hypoxylon anthochroum* (KF192825)	100	KY985428
*Rhizophora mucronata*	*Roccella montagnei*	KO4_22	*Lasiodiplodia crassispora* (KP724987)	100	MF029743
		KO4_34	*Daldinia eschscholtzii* (FJ624265)	100	MF029744
*Rhizophora mucronata*	*Dirinaria picta*	KO5_30	*Daldinia eschscholtzii* (FJ624265)	100	MF029746
		KO5_33	*Daldinia* sp. (KC343208)	100	MF773665
		KO5_8	*Daldinia eschscholtzii* (KY432354)	100	MF773671
		KO5_12	*Hypoxylon anthochroum* (KF192825)	99	MF773672
*Clerodendron inerme*	*Opegrapha medusulina*	KO6_12	Xylariaceae sp. (AB440126)	100	MF029748
		KO6_17	*Schizophyllum commune* (LT217536)	100	MF773673
*Thesphesia populnea*	*Porina tetracerae*	KO7_26	*Endomelanconiopsis endophytica* (EU683656)	100	MF029750
		KO7_2	*Daldinia eschscholtzii* (FJ624265)	100	MF773674
		KO7_18	*Aspergillus aculeatus* (KX098322)	100	MF773675
		KO7_30	*Talaromyces* sp. (KF225854)	99	MF773676
*Rhizophora mucronata* *Rhizophora mucronata*	*Porina tetracerae* *Porina tetracerae*	KO8_8	*Endomelanconiopsis* sp. ( KU997686)	100	MF029751
		KO8_19	*Phomopsis* sp. (DQ780433)	99	MF773677
*Aegiceras corniculatum*	*Pyrenocarp* sp.	KO9_10	*Cerrena* sp. (KX911717)	100	MF773678
*Excoecaria agallocha*	*Pyrenula indet*	KO10_6	*Endomelanconiopsis endophytica* (FJ799942)	100	MF773679
		KO10_6ST	*Endomelanconiopsis* sp. (KU747787)	100	MF029752
		KO10_9	*Cerrena* sp. ( KX599411)	100	MF029753
*Aegiceras corniculatum*	Unidentified sterile sp.4	KO11_26	*Trichoderma harzianum* (KR868236)	100	MF029755
		KO11_11	*Lasiodiplodia pseudotheobromae* (KP724987)	100	MF029754
		KO11_7	Sordariomycetes (KM519240)	99	MF773680
		KO11_8	*Nigrospora sphaerica* (KC881195)	100	MF773681

### Extraction of genomic DNA and polymerase chain reaction (PCR)

All isolates were inoculated into Potato Dextrose Broth (PDB) and incubated at room temperature (30–32°C) for 7 days. The mycelia were separated from the broth and the DNA was extracted using the modified method described in Cenis (1992) [18]. Quality of the extracted DNA samples was tested using 1% agarose gel electrophoresis and samples were stored at -20 ^o^C until use. Fungal rDNA-ITS region was amplified from the purified genomic DNA by using the fungal specific ITS1 and ITS4 universal primers [19]. The PCR reaction mixture comprised of 2–5 μL fungal DNA, 1 × PCR buffer, 1.5 mM MgCl_2_, 0.2 mM each dNTP, 1μM each forward and reserve primer, 1.25 units of Taq DNA polymerase (Promega, USA) as described in Attanayake et al. (2009) [20]. PCR protocol was as follows, an initial denaturation step at 94°C for 5 min, followed by 30 cycles of 94°C for 30 sec, 55°C for 30 sec, and 72 ºC for 30 sec, with a final extension step of 72 ºC for 10 min. PCR was performed in a Veriti™ Thermal Cycler (Applied Biosystems, Foster City, CA, USA). The amplification of ITS region was confirmed by separating the amplified product in 1% agarose containing 0.2–0.5 μg/mL ethidium bromide using gel electrophoresis. Single PCR products were directly sequenced using Sanger dideoxy sequencing technology at Genetech, Colombo, Sri Lanka.

DNA sequences were manually edited using BioEdit program [21] and compared with the sequences available in the GenBank using Basic Local Alignment Search Tool (BLAST). Microscopic characters of the pure cultures were in combination with the molecular data were used for ultimate species delineating as described in Attanayake et al. (2009) [20].

DNA sequences of the identified ELFs species were deposited at the NCBI database and accession numbers obtained for future reference (S1 File). ELFs identified in this study were shown in the Table 1 with the percent similarity to the published/authenticated sequences at the GenBank.

### Phylogenetic analysis of ELFs

DNA sequences were aligned by using a multiple sequence alignment algorithm, Multiple Sequence Comparison by Log- Expectation (MUSCLE) [22]. GBlocks 0.91b was used to eliminate poorly aligned positions and divergent regions of the alignment [23]. All characters were equally weighted and the gaps were treated as missing data. Sequence of ITS region of a Zygomecete, *Mortierella elongata* (AB542112.1), was used as the out group. The evolutionary history was inferred by using several phylogenetic tree construction methods, maximum likelihood, maximum parsimony and neighbor-joining, implemented in MEGA ver. 7.0 software. For Maximum Likelihood method, initial tree(s) for the heuristic search were obtained automatically by applying Neighbor-Join and BioNJ algorithms to a matrix of pair-wise distances estimated using the Maximum Composite Likelihood (MCL) approach, and then selecting the topology with superior log likelihood value. One thousand boot strap replications were used as statistical support for the nodes in phylogenetic trees using MEGA [24].

### Extraction of secondary metabolites

All the endolichenic fungi were cultured separately on PDA for 2 weeks. The mycelium of each fungus and the medium were cut into small pieces, extracted with EtOAc (6 × 500 mL) and the solvent of each extract were evaporated under reduced pressure. The EtOAc extracts were transferred to glass vials separately and passed N_2_ through the samples to remove remaining solvent. The resulting semisolid extracts were stored at 0°C until use for the bioassays [9].

### Radical scavenging ability by 2, 2-diphenyl-1-picrylhydrazyl (DPPH) method

The radical scavenging ability assay was carried out in a flat bottom 96-well microtiter plate, according to the method described by Chatatikun and Chiabchalard (2013) [25] with slight modifications. Different doses of each test sample and standard antioxidant, Butylated hydroxytoluene (BHT) (12.50, 25.00, 50.00, 100.00, 200.00, 400.00, 800.00 μg/mL) was added to 40 μL of 0.25 mM methanolic DPPH solution in the 96-well plate. All reagents were mixed and incubated for 15 min at room temperature under dark conditions. The absorbance of each well was measured at 517 nm with a Microplate Reader (Biotek, USA) (S2 File). The percentage inhibition was calculated using the Eq 1. The IC_50_ values of all the crude extracts were calculated using Prism 7 Release 2017, Statistical Software. The experiment was carried in triplicates.

$$\%inhibition=\frac{control-sample}{sample}\times 100$$ (1)

### Antilipase assay

Lipase inhibitory assay was designed to evaluate anti-obesity property of each endolichenic fungal extract. The assay was carried out in a flat bottom 96-well microtiter plate, according to the method described by Gilham et al. (2004) [26] with slight modifications. Each ELF extract and the positive control (100 μg/mL) was pre-incubated with 50 μL of lipase enzyme (1mg/mL in tris buffer, pH 7.4) for 10 min and 50 μL of p-nitrophenyl acetate (4 mM in tris buffer, pH 8.0) was added to the test sample separately. The mixture was further incubated at 37°C for 30 min. Control and blank samples were prepared without test samples or lipase enzyme separately. The absorbance was measured at 405 nm and Orlistat was used as positive control (S3 File). The percentage of inhibition of lipase activity was determined using the Eq 1. The experimnet was carried in triplicates.

### Alpha amylase inhibition assay

The α-amylase assay was also carried out in a flat bottom 96-well microtiter plate, according to the method described by Bhutkar et al. (2012) [27] with slight modifications. The determination of α-amylase inhibition was carried out by quantifying the reducing sugar (maltose equivalent) released under the assay conditions. The enzyme inhibition was stated as a reduction in units of maltose liberated. The dose of each test sample was 250 μg/mL to screen α-amylase inhibition and the extracts were pre-incubated with 100 μL of α-amylase (1 U/mL) for 10 min separately and thereafter 100 μL (1% w/v) starch solution was added. The mixture was further incubated at 37°C for 10 min. Further the reaction was stopped by adding 100 μL of DNS reagent (12.0 g of sodium potassium tartrate tetrahydrate in 8 mL of 2 M NaOH and 96 mM 3, 5- dinitrosalicylic acid solution) and the contents were heated in a boiling water bath for 10 min. The blank samples were prepared without test sample or the amylase enzyme separately; and that volume was replaced by equal quantities of buffer (20 mM Sodium phosphate buffer with 6.7 mM Sodium chloride, pH 6.9). Moreover, acarbose was used as the positive control. The inhibition of α-amylase was calculated using the Eq 1 to evaluate the antidiabetic activity of each extract after recording of absorbance at 540 nm (S4 File). The experiment was carried out in triplicates.

### Statistical analysis

The data were presented as the mean ± standard error of the mean (SEM). IC_50_ values were calculated by using Graph Pad Prism (Verson 6.0) software package. Differences between samples were considered significant at P < 0.05 and calculated by using Minitab-17. Principal Component Analysis (PCA) and correlation analysis were carried out to determine the correlation of bioactivities by the endolichenic fungi.

## Results and discussion

### Identification of lichens

Sri Lanka harbors a diverse flora and fauna including many endemic plant species and this is also true with regard to the lichens. It is reported that nearly 1200 lichen species are known from Sri Lanka [28]. The literature survey revealed that only a very few studies on isolation, identification and bioprospecting properties of endolichenic fungi had been reported. In the present study, the total number of lichen species collected in the study areas were 32 from four sites (Figs 1 and 2). Of the 32 lichen samples, 18 were on the mangrove plants while the rest were collected from mangrove associated plants (Table 1). The distinctive morphological characters and the molecular identification of all the lichen samples revealed that they are previously known species suggesting that the biodiversity of mangrove lichens in Puttalam lagoon of Sri Lanka is limited. It should be noted that these lichens were limited to few mangrove and mangrove associated plant species in the study area (Table 1).

### Isolation and identification of ELF

Total of 171 fungal isolates were isolated from 32 lichen samples. Isolates were identified up to the genera using morphological characteristics. Species confirmation was achieved by comparing sequence of ITS rDNA region with the established (voucher specimens or published) records with the highest percent similarity of the GenBank accessions and are shown in the Table 1. The table shows host plant species, names of the lichens, name of each ELF isolate and their accession numbers and the GenBank accession numbers of the sequences that showed the highest similarity (S5 File). It was found that ninety percent of the isolates belonged to the phylum Ascomycota while the rest belonged to the phylum Bascidiomycota and this finding is in line with previous endolichenic fungal diversity studies [29–31]. Members of Phylum Ascomycota comprised of 11 families whereas phylum Bascidiomycota comprised only three families. Though Zhang et al (2016) reported the presence of endolichenic fungal species belonging to phyla Ascomycota, Basidiomycota, and Zygomycota in lichen samples obtained from Arctic ecosystems, no member belonged to the phylum Zygomycota in the current study. However, it is important to note that Zhang et al. (2016) used 454-Next Generation sequencing platform in which both culturable and unculturable species are recovered and in the current study only culturable isolates were studied [31].

### Phylogenetic analysis of ELF

**Fig 4:**
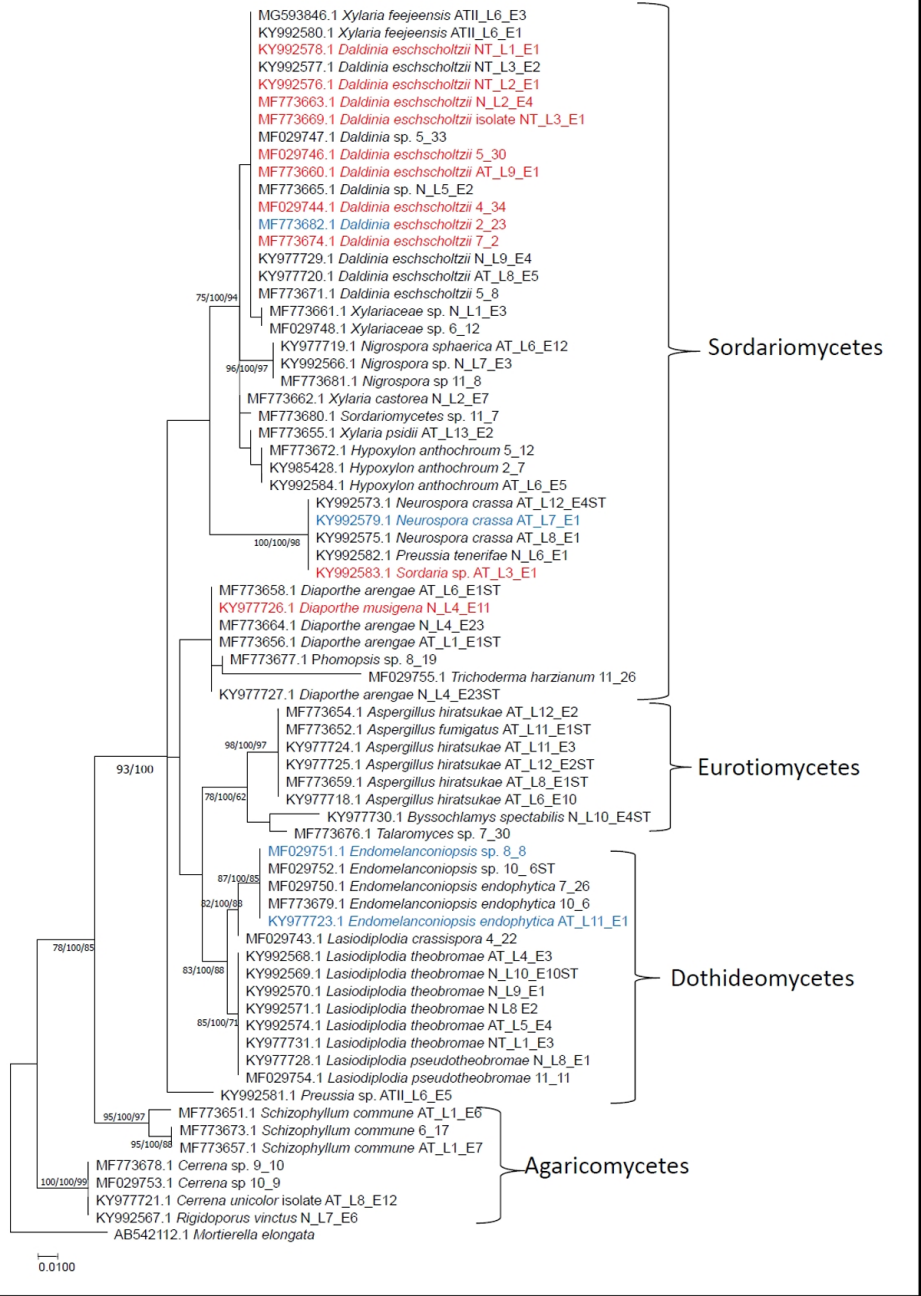
Maximum likelihood tree with the highest log likelihood showing phylogenetic placement of 70 endolichenic fungi isolated from lichens in the mangrove and mangrove associated plants in Puttalum lagoon, Sri Lanka. The tree is drawn to scale, with branch lengths measured in the number of substitutions per site. Numbers near each node represent the bootstrap support obtained in Maximum Likelihood, Maximum Parsimony and Neighbor Joining methods respectively.

Multiple sequence alignment (MSA) was done with approximately 500–600 length sequences. All positions containing gaps and missing data were eliminated and there were a total of 178 positions in the final dataset. Since the choice of phylogenetic analysis method did not have a major impact on tree topology, only Maximum Likelihood tree with the highest log likelihood (-813.33) is shown in Fig 4. The tree was drawn to scale, with branch lengths measured in the number of substitutions per site. The percentage of replicate trees in which the associated species clustered together in the bootstrap test in all three methods were shown on top of the branches [32]. In all three analyses, isolates were clearly grouped according to families and genera. Seventy isolates belonged to 18 genera, *Aspergillus* (6), *Byssochlamys* (1), *Cerrena* (3), *Diaporthe* (5), *Daldinia* (15), *Endomelanconiopsis* (5), *Hypoxylon* (3), *Lasiodiplodia* (9), *Neurospora* (3), *Nigrospora* (3) *Preussia* (2), *Phomopsis* (1), *Rigidoporus* (1), *Schizophyllum* (3), *Sordaria/Sordariomycete* (2), *Talaromyces* (1), *Trichoderma* (1) and *Xylaria* (6) as indicated in the ML tree. Similar to the study conducted by Arnold et al. (2009) most of the endolichenic fungi belonged to families Sodariomycetes and Dothdeomycetes. In the phylogenetic tree, fungal species clustered together regardless of their lichen host indicating that there is little or no role played by the lichen host on selecting their particular endolichenic fungal species [29, 33].

Isolates labelled in red color had high antioxidant activities and blue color had potential anti lipase activity. When both colors are shown it indicates that the same isolate had both activities.

Of the 32 lichen samples, the highest number of endolichenic fungal species (05) was identified from the lichen thallus of *Opegrapha medusulina* (AT-06) and an unidentified sterile lichen species (AT-11). The lichens, *Amandinea medusulina* (AT-13), *Arthonia parantillarum (*NARA-06), *Arthonia antillarum (*AT-04), *Arthonia* sp. (NARA-01), *Opegrapha medusulina* (AT-02, AT-09, NT-02 and NT-03), *Pyrenula parvinuclea (*AT-05 & AT-07), *Pyrenula parvinuclea* (AT-07), *Pyxine cocoes (*NARA-05), *Roccella montagnei (*NARA-02), and Sterile *Pyrenocarp* sp. *(*KO-09), yielded a single endolichenic fungal species in each. The genera, *Lasiodiplodia* and *Daldinia* were the most common ELF constituting 32.3% of the total isolates.

Only selected isolates were used in bioassays. Therefore, bioassays were conducted for 70 isolates. Nearly 38.2% of the total endolichenic fungal isolates were considered as fungi that can produce bioactive compounds as some of the ELF showed positive results for radical scavenging activity or antilipase activity.

### Screening of radical scavenging activity

**Fig 5:**
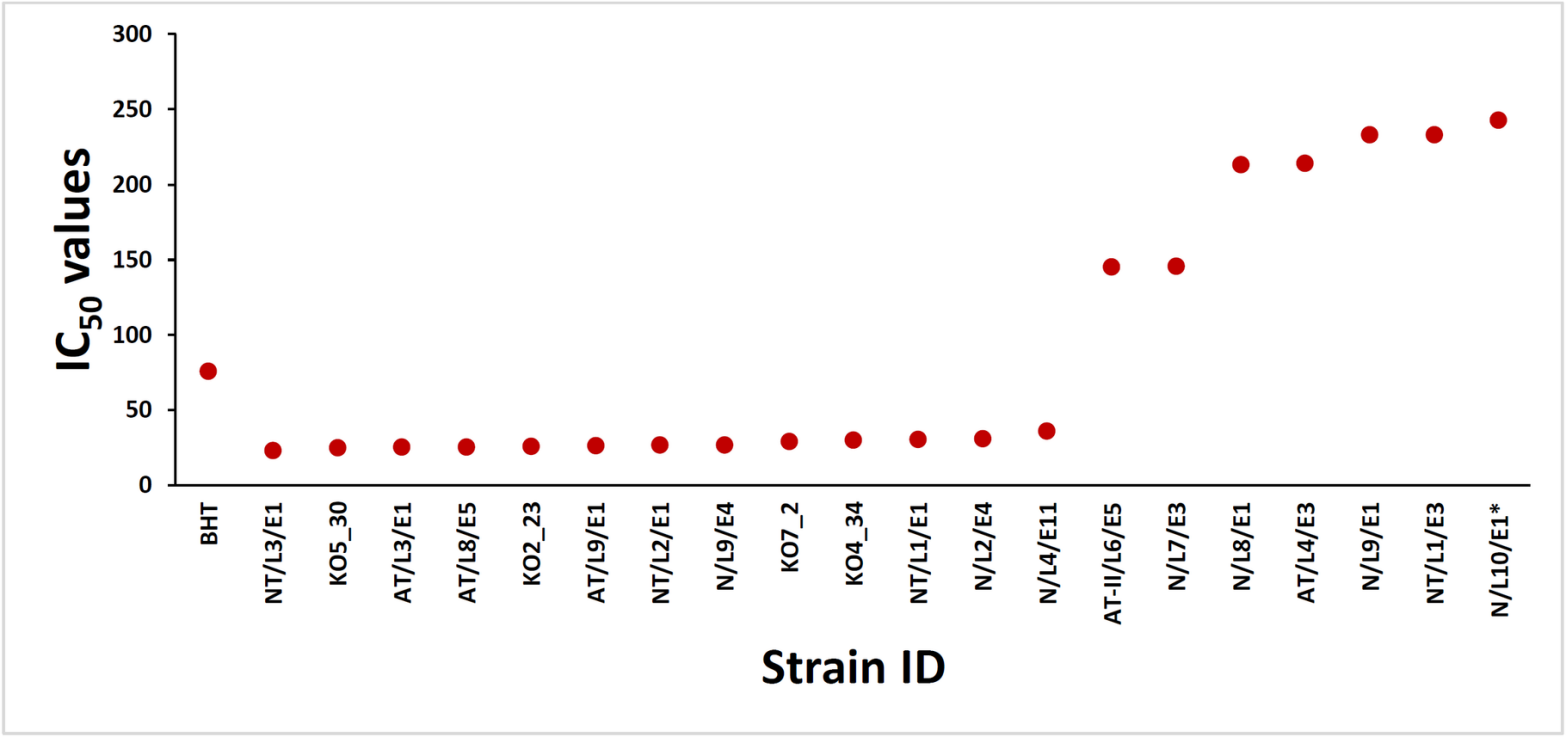
IC_50_ values (μg/mL) of crude ethyl acetate extracts of the endolichenic fungi recovered from lichens on mangrove and mangrove associated plats in Puttalam lagoon *Foot note*: Three replicates were used for each fungal extract and IC_50_ values (μg/mL) were calculated by using Graph Pad Prism (Verson 6.0) software package. Note: Fungal extracts with IC_50_ values below 243 μg/mL are presented in the graph.

**Fig 6:**
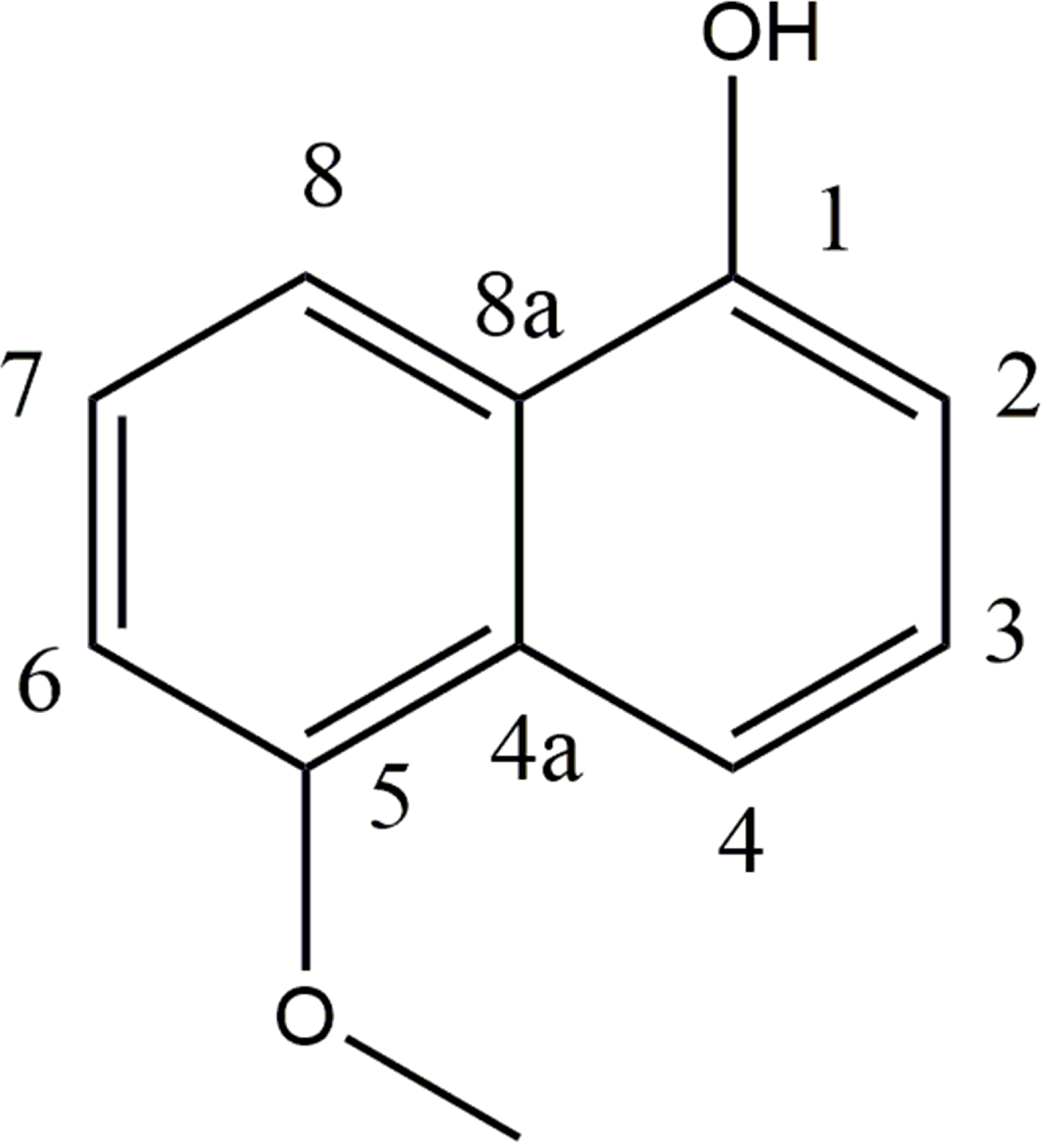
Bioactive compound isolated from the endolichenic fungus, *D*. *eschscholtzii* isolated from the lichen, *Parmotrema* sp. collected from Hakgala Botanical Garden in Sri Lanka.

Antioxidants are extremely important constituents, which possess the ability to protect the human body from non-communicable diseases and prevent the damage to the tissues caused by free radical induced oxidative stress. The antioxidant potential of the ELF isolated from mangrove and mangrove associated plants were investigated in the search for new bioactive compounds from natural resources. The LC_50_ values obtained for DPPH radical scavenging activities of the ELF are presented in Fig 5 and Table 2. Extracts of *D*. *eschscholtzii* and *Sordaria* sp. isolated from different lichens recorded the highest radical scavenging activity against DPPH assay with IC_50_ values ranging from 24.6 μg/mL– 31.19 μg/mL. Second best activity was reported with *Diaporthe musigena* obtained from *Arthonia antillarum* with LC_50_ value 37.27 μg/mL and these IC_50_ values were significantly different (P<0.05) from the +ve control, BHT (IC_50_ 76.5 27 μg/mL). Strain specificity for bioactivity was strongly observed. For example *D*. *eschscholtzii* obtained from 10 of the lichens, *Roccella montagnei* from *Cocos nucifera* (mangrove associated) and *Rhizophora mucronata*, unidentified Sterile sp. 2 from *Soneratia* sp., *Opegrapha medusulina* from *Cocos nucifera*, *Opegrapha arabica*, *Opegrapha medusulina* from *Excoecaria agallocha*, *Crateria dissimilis* from *Rhizophora mucronata*, *Dirinaria picta* from *Rhizophora mucronata*, *Arthonia* sp. from *Thesphesia populnea* showed IC_50_ values which are not significantly different from each other (P>0.05) indicating similar secondary metabolites may be present in these fungal strains. The bioactive compound of the ELF, *D*. *eschscholtzii* isolated from the lichen, *Parmotrema* sp. collected from Hakgala Botanical Garden in Sri Lanka was identified as 5-methoxynaphthalen-1-ol (Fig 6) and this compound showed very high radical scavenging activity with IC_50_ 10.2 μg/mL when compared to the positive control, BHT [34]. The endolichenic fungi, *Xylariaceae* sp., *Aspergillus aculeatus*, *Talaromyces* sp., *Endomelanconiopsis* sp., *Endomelanconiopsis* sp., *Nigrospora sphaerica*, revealed very poor antioxidant activity as the percentage inhibition obtained for the highest concentration tested were less than 50%. Isolation of bioactive compound with antioxidant activity have been reported before. The IC_50_ value of ethyl acetate extract of an ELF, *Talaromyces* sp. had been reported as 45.50±0.01 against DPPH assay [25] and isolation of two novel polyketides from an ELF, *Penicillium citrinum*, with potent radical scavenging activity (IC_50_ values of 159.7 ± 22.3 μg/mL and 68.6 ± 4.3 μg/mL) in DPPH antioxidant assay were reported [8]. Hence, the present study confirmed that ethyl acetate extracts of the ELF contain secondary metabolites with significantly high radical scavenging activities (P < 0.05). It is confirmed that the scavenging effects of the ELF extracts with lower IC_50_ values (< 50) were the excellent sources to isolate bioactive compounds with proton-donating ability and could serve as free radical inhibitors or scavengers, acting possibly as primary antioxidants.

**Table 2:** Radical scavenging, amylase inhibition and lipase inhibition activities of ethyl acetate extracts of the endolichenic fungi isolated from mangrove and mangrove associated plants from Puttalam lagoon in Sri Lanka.

Name of the host plant	Name of the lichen	Name of the ELF	IC_50_ Value of DPPH assay (μg/mL)	% inhibition at 250 μg/mL of test compound for α-amylase	% inhibition at 100 μg/mL mL of test compound for lipase
*Aegiceras corniculatum*	Unidentified Sterile sp. 1	*Schizophyllum commune* (MF773651)	325.60±0.58lmn	2.69±0.11c	3.79±1.00n
		*Diaporthe arengae* (MF773656)	375.90± 0.79j	1.60±0.26c	13.18±0.68g
		*Schizophyllum commune* (MF773657)	330.20±0.42klm	3.01±0.10c	4.56±1.10n
*Soneratia* sp.	*Opegrapha medusulina*	*Aspergillus hiratsukae* (MG593848)	295.60±6.15op	0.35±0.40b	2.56±0.40ab
*Soneratia* sp.	Unidentified Sterile sp. 2	*Sordaria* sp. (KY992583)	26.03±0.41v	0.61±0.07b	7.24±0.23cdef
*Thespesia populnea(Mangrove associated)*	*Arthonia antillarum*	*Lasiodiplodia theobromae* (KY992568)	211.20± 1.09r	11.42±1.48d	12.99±0.98n
*Thespesia populnea* (Mangrove associated)	*Pyrenula parvinuclea*	*Lasiodiplodia theobromae* (KY992574)	260.60±9.36r	11.66±1.40d	14.94±1.73n
*Rhizophora neucronuta*	*Opegrapha medusulina*	*Hypoxylon anthochroum* (KY992584)	851.4±0.31v	10.50±2.53d	10.00±0.83fg
		*Aspergillus hiratsukae* (KY977718)	301.20±6.15op	0.25±0.01b	3.27±0.25n
		*Nigrospora sphaerica* (KY977719)	423.30±1.76gh	0.98±0.10b	16.22±0.88h
		*Diaporthe arengae* (KY977727)	336.30±17.94j	3.01±0.26	14.01±0.29h
		*Xylaria feejeensis* (MG593846)	MI <50%	7.61±0.41d	19.71±0.97j
		*Chaetomium fuscum* (KY992580)	456.30± 1.20fg	0.20±0.04b	2.89±1.25ab
		*Preussia* sp. (KY992581)	147.0±2.56t	0.86±0.09b	14.57±0.70h
*Cocos nucifera* (Mangrove associated)	*Pyrenula parvinuclea*	*Neurospora crassa* (KY992579)	281.5±1.0nop	8.23±2.31d	34.87±5.13lm
*Cocos nucifera* (Mangrove associated)	*cf*. *Roccella montagnei*	*Neurospora* sp. (KY977728)	341.36±1.83jkl	11.05±1.53d	29.50±4.47ijklm
		*Daldinia eschscholtzii* (KY977720)	25.91± 0.48v	2.33±0.24b	28.86±2.92klm
		*Cerrena unicolor* (KY977721)	426.30± 1.49gh	1.44±0.09b	1.62±0.51a
*Cocos nucifera* (Mangrove associated)	*Opegrapha medusulina*	*Daldinia eschscholtzii* (MF773660)	27.81±2.41v	1.29±0.27b	39.72±1.86n
*Cocos nucifera* (Mangrove associated) *Cocos nucifera* (Mangrove associated)	Unidentified Sterile sp. 3	*Lasiodiplodia theobromae* (MG593847)	250.50± 1.05r	10.53±1.87d	15.84±0.82n
		*Endomelanconiopsis endophytica* (KY977723)	526.7±1.76b	8.21±0.67d	30.76±4.04ijklm
		*Aspergillus hiratsukae* (KY977724)	465.20±2.15e	1.99±0.34c	6.90±1.55n
*Cocos nucifera*(Mangrove associated)	*Pyxine cocoes*	*Aspergillus hiratsukae* (KY992573)	474.6±0.32ef	6.76±1.39c	5.62±0.65cd
		*Neurospora crassa* (KY992573)	304.5±1.03pq	1.57±0.08c	37.71±2.31m
*Cocos nucifera(Mangrove associated)*	*Amandinea medusulina*	*Xylaria psidii* (KY992572)	361.04± 0.86j	1.32±0.14c	9.23±0.65defg
*Excoecaria agallocha*	*Opegrapha arabica*	*Daldinia eschscholtzii* (KY992578)	31.42±0.57v	1.72± 0.05d	14.76±0.30h
		*Lasiodiplodia theobromae* (KY977731)	230.6± 1.92r	11.42±1.78d	10.86±1.64g
*Excoecaria agallocha*	*Opegrapha medusulina*	*Daldinia eschscholtzii* (KY992576)	26.11±2.41v	1.84±0.54c	31.20±1.60n
*Excoecaria agallocha*	*Opegrapha medusulina*	*Daldinia eschscholtzii* (MF773669)	23.81±2.41v	2.36±1.19c	27.76±0.70n
*Salvadora persica*	*Arthonia* sp	*Xylariaceae* sp. (MF773661)	MI <50%	0.69±0.02b	24.67±2.16ijkl
*Syzygium* sp.	*cf*. *Roccella montagnei*	*Daldinia eschscholtzii* (MF773663)	31.19±0.57v	0.99±0.01b	26.44±2.46
		*Xylaria castorea* (MF773662)	420.30±1.41gh	0.01±0.00b	7.65±0.92cdef
*Syzygium* sp.	*Arthonia antillarum*	*Diaporthe musigena* (KY977726)	37.27±1.019v	1.46±0.11c	10.21±0.21fg
		*Diaporthe arengae* (KY977727)	365.90±17.94j	2.08±0.36c	12.20±0.15g
*Syzygium* sp.	*Pyxine cocoes*	*Daldinia* sp. (MF773665)	430.0±0.75g	1.40±0.07c	22.61±1.24ijk
*Cocos nucifera* (Mangrove associated)	*Arthonia parantillarum*	*Preussia tenerifae* (KY992582)	312.60±0.34mno	2.95±0.22c	22.91±1.06ijk
*Cassia auriculata* (Mangrove associated)	*cf*. *Roccella montagnei*	*Lasiodiplodia theobromae* (KY99257)	256.31± 0.44r	11.36±1.41d	8.62±1.13n
		*Lasiodiplodia pseudotheobromae* (KY977728)	212.50±0.85i	10.87±0.75d	14.05±0.12h
*Cocos nucifera(Mangrove associated)*	*cf*. *Roccella montagnei*	*Lasiodiplodia theobromae (KY992570)*	231.50± 0.72r	11.86±1.78d	6.88±0.46n
		*Daldinia eschscholtzii* (KY977729)	29.15±0.56v	3.02±0.05c	25.80±2.26n
*Cassia auriculata* (Mangrove associated)	*Pyrenula ochraceoflava*	*Lasiodiplodia theobromae* (KY977730)	241.10±9.36r	13.01±1.15d	8.17±0.55n
		*Byssochlamys spectabilis* (KY992570)	421.32±1.07gh	1.53±0.37b	2.57±0.41ab
		*Chaetomium* sp. (KY977729)	476.40±2.08ef	0.523±0.11b	3.21±0.28ab
*Rhizophora mucronata*	*Crateria dissimilis*	*Daldiniae schscholtzii* (MF773682)	25.71±2.41v	1.35±1.00c	30.56±0.71
		*Hypoxylon anthochroum* (KY98542)	306.30±0.96mno	12.23±0.77d	22.57±2.96ijk
*Rhizophora mucronata*	*Crateria dissimilis*	*Lasiodiplodia crassispora* (MF029743)	396.23±1.66i	12.04±0.78d	8.62±0.56fg
		*Daldinia eschscholtzii* (MF029744)	29.51±0.56v	2.50±1.00c	27.96±0.70n
*Rhizophora mucronata*	*Lenticells only*	*Daldinia eschscholtzii* (MF029746)	24.60±2.41v	1.77±0.06c	23.75±1.53n
		*Nodulisporium* sp.(MF773671)	523.26± 2.05d	2.95±0.64c	19.16±3.09ij
		*Hypoxylon anthochroum* (MF773672)	675.63±0.704a	11.67±1.01d	13.79±1.03h
*Thesphesia populnea*	*Arthonia* sp.	*Endomelanconiopsis endophytica* (MF029750)	676.50±1.03d	6.80±0.20d	37.34±1.79n
		*Daldinia eschscholtzii* (MF773674)	29.23±0.56v	1.96±0.56c	28.09±1.33n
		*Aspergillus aculeatus* (MF773675)	MI <50%	4.54±1.02c	15.45±0.63h
		*Talaromyces pinophilus* (MF773676)	MI <50%	0.43±0.01b	4.20±0.27b
*Rhizophora mucronata Rhizophora mucronata*	*Porina tetracerae Porina tetracerae*	*Endomelanconiopsis* sp. (MF029751)	MI <50%	9.33±0.70d	32.59±2.28klm
		*Phomopsis* sp. (MF773677)	432.51±2.32g	4.48±1.33c	15.23±0.89h
*Aegiceras corniculatum*	*Sterile Pyrenocarp specimen*	*Cerrena* sp. (MF773678)	361.32±1.70i	2.30±0.43c	2.31±0.45n
*Exoecaria aggalocha*	*Pyrenula indet*	*Endomelanconiopsis* sp. (MF773679)	MI <50%	3.44±0.59c	9.45±0.6efg
		*Cerrena* sp. (MF029753)	676.50±1.40c	1.69±0.97c	3.29±0.64ab
*Aegiceras corniculatum*	Unidentified Sterile sp. 4	*Trichoderma harzianum* (MF029755)	403.30±2.26hi	2.24±0.56c	1.56±0.74a
		*Lasiodiplodia pseudotheobromae* (MF029754)	390.50±2.2s	9.21±0.78d	11.63±0.72g
		*Sordariomycetes* sp. (MF773680)	350.61±1.21jk	0.87±0.07b	1.32±0.31a
		*Nigrospora sphaerica* (MF773681)	MI <50%	0.46±0.03b	5.70±0.42cd
			Acarbose (+ve Control) 55.274±4.13a	Orlistat (+ve Control) 62.60±4.18p	BHT (+ve Control) 76.50±1.47u

The letters, a—v represent significant differences of endolichenic fungi against DPPH assay, antilipase assay and amylase inhibition assay (*p* < 0.05). ELF–Endolichenic fungi, IC_50_−50% inhibitory concentration, DPPH– 2, 2-diphenyl-1-picrylhydrazyl, BHT—Butylated hydroxytoluene, MI–minimum inhibition

### Antilipase assay

**Fig 7:**
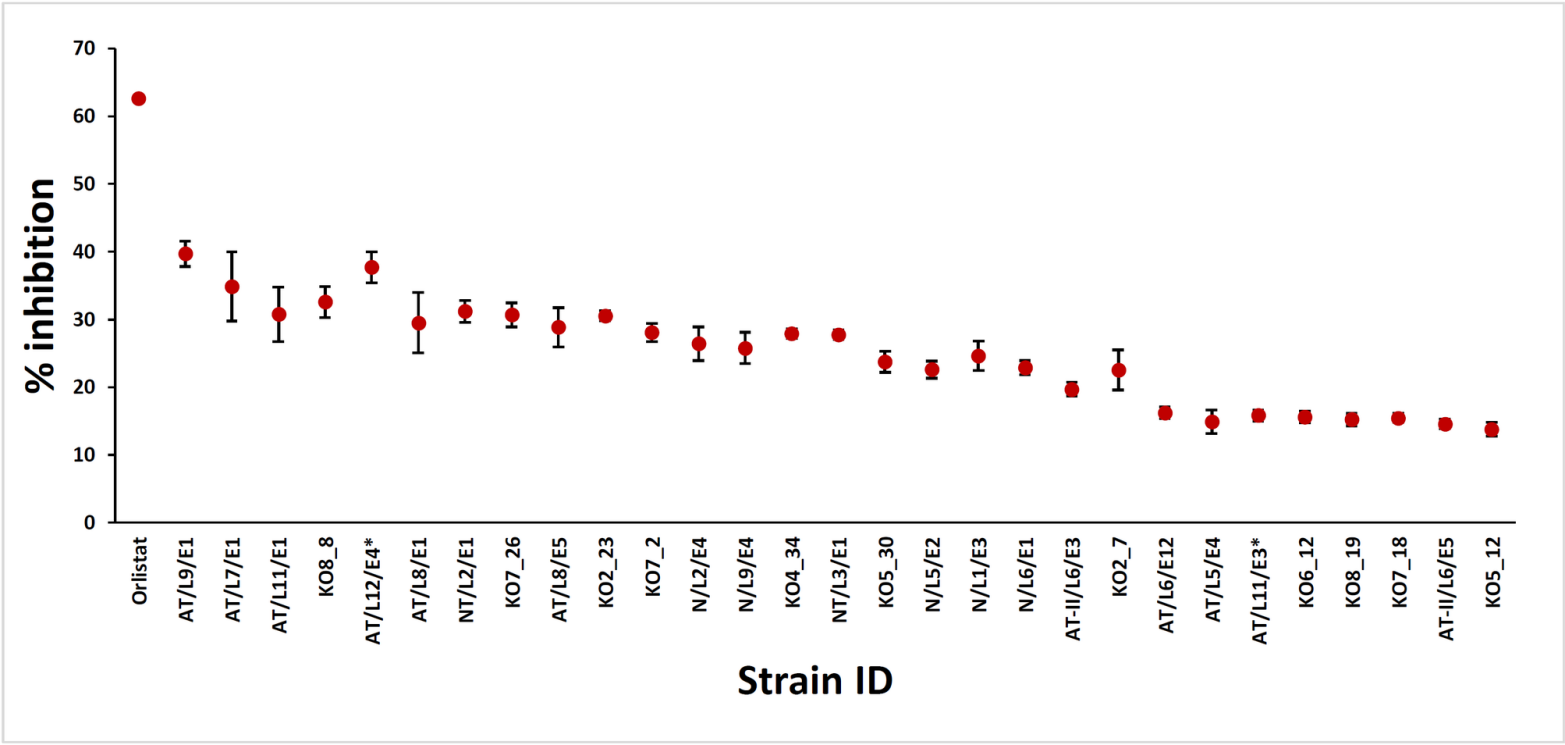
Antilipase activity of crude ethyl acetate extracts of endolichenic fungi recovered from lichens on mangrove and mangrove associated plants in Puttalam lagoon. *Footnote*: three replicates were used for each fungal extracts and fungal extracts with % inhibition above 14 are presented in the graph.

Obesity is associated with non-communicable diseases and it becomes the fifth leading risk of global deaths (http://easo.org/education-portal/obesity-facts-figures). World Health Organization (WHO) reports that 13% of total global population is obese and Orlistat is the only prescribed drug approved for the long term treatment of obesity (http://www.who.int/mediacentre/factsheets/fs311/en). Hence there is an urgent need to investigate alternative drugs for Orlistat from natural sources. It is reported that fungi also offer to be a promising underexplored resource for screening potential lipase inhibitors. Antiobesity activity of ethyl acetate extract of endophytic fungi had been reported and the results revealed that an endophytic fungus, *Penicillium* sp. isolated from *Taxus baccata* showed promising activity with an IC_50_ of 3.69 μg/ml [35]. In the present study, inhibition of lipase enzyme was carried out in a flat bottom 96-well microtiter plate, using the method described by Gilham et al. (2005) [26] with slight modifications. Percentage inhibition of the lipase enzyme was calculated at the dose of 100 μg/mL and presented in Table 2 and Fig 7. The results were compared with the positive control, Orlistat which showed the highest percentage inhibition (62.6%). Of the extracts of ELF, the highest activity was observed in *Endomelanconiopsis endophytica* (37.34%) isolated from *Arthonia* sp., nevertheless this antilipase activity was significantly different from the positive control (P < 0.05). Results of the antilipase assay revealed that only fifteen extracts exhibited the percentage inhibition above 25% and those extracts can be considered when isolating bioactive compounds against obesity. Isolation of antiobesity compounds had been reported from an endolichenic fungus, *Nodulisporium* sp. (No. 65-17-2-1) fermented with potato-dextrose broth and eight new compounds, nodulisporiviridins A-H have been isolated with potent antiobesity activity [36].

### Alpha-amylase inhibition assay

**Fig 8:**
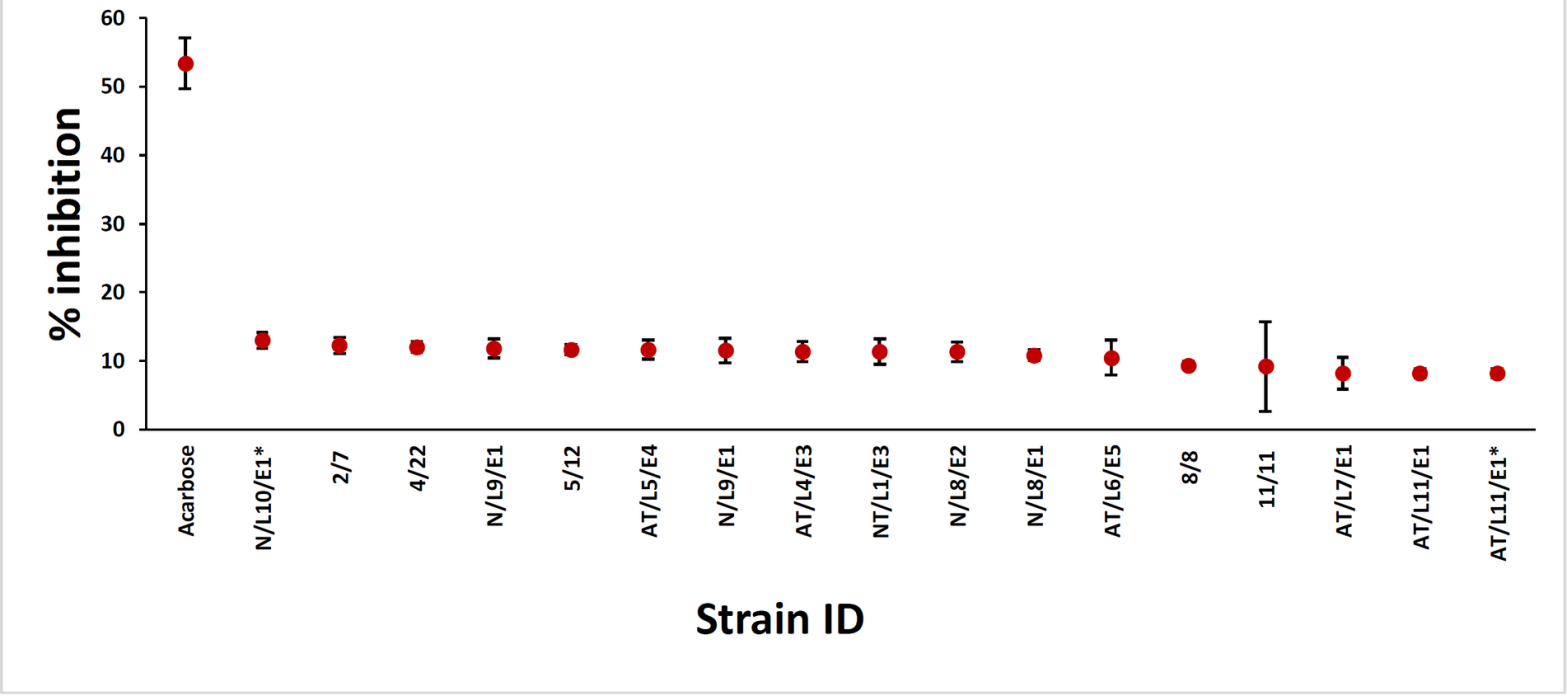
Alpha amylase inhibition activity of crude ethyl acetate extracts of endolichenic fungi recovered from lichens on mangrove and mangrove associated plants in Puttalam lagoon. *Footnote*: three replicates were used for each fungal extract and fungal extracts with % inhibition above 8% are presented in the graph.

Diabetes mellitus is one of the common non-communicable diseases and around 2.8% of the world suffers from diabetes and is expected to increase 5.4% by the year 2025. Endolichenic fungi are reported to produce a large number of bioactive secondary metabolites and serve as an excellent source of drugs for treatment of metabolic disorders. However, a thorough literature survey on antidiabetic compounds from endolichenic fungi reveals that dearth of information on isolation of antidiabetic compounds from endolichenic fungi is available. Therefore, inhibition of α-amylase enzyme by extracts of the ELF was investigated using the method described by Bhutkar et al. (2012) [27] with slight modifications. The results revealed that none of the ELF fungi exhibited the potent inhibition except *Lasiodiplodia theobromae* which showed 13% inhibition at 250 μg/mL as compared with positive control Acarbose which recorded 55.3% inhibition (Fig 8 and Table 2). The present study is a pioneer work wherein we have explored the potential amylase inhibitory activity of endophytic fungi which were isolated from different mangrove lichens. The results indicate that high activity of the enzyme inhibition was not found in the ELF fungi reported in this study. Hence, till date there exists no report on endolichenic fungi producing an amylase inhibitor.

**Fig 9:**
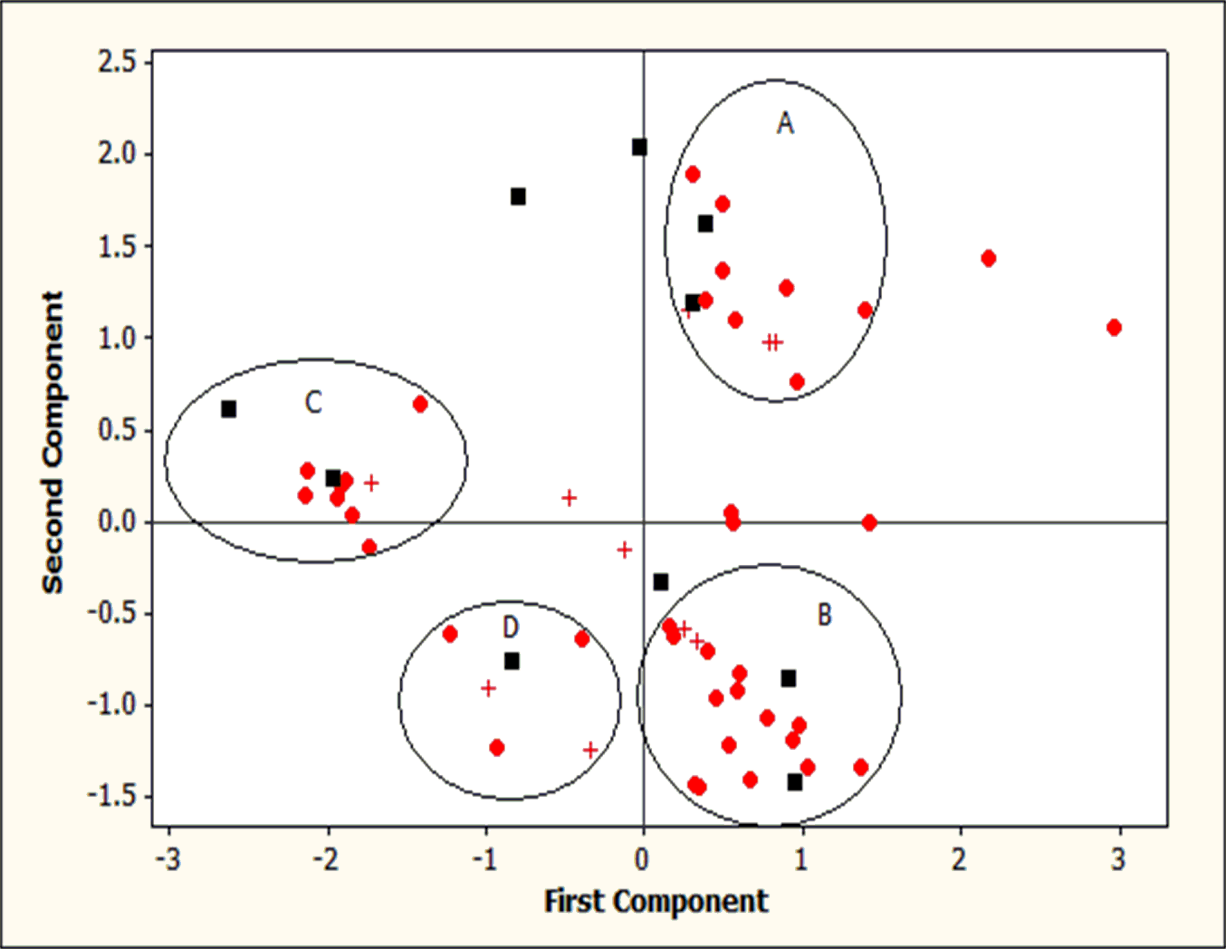
Principal component analysis (PCA) of endolichenic fungi from lichens in mangroves and mangrove associated plants. PCA showing the correlation between different endolichenic fungi and their biological activities against DPPH assay, antilipase assay and amylase inhibition assay. *Daldinia* sp. and *Lasiodiplodia* sp. were shown in square and cross marks respectively.

PCA analysis improved the visual interpretation of data. First two components explained 81.8% of the variation. While the variance of anti-lipase and alpha amylase inhibition assays were best described by the principle component (PC) 2, the variance of free radical scavenging capacity was best described by the PC 1 (S1 File). Since the highest number of fungal species reported was *Daldinia* sp. and *Lasiodiplodia* sp. those two were marked differently in the Fig 5. Based on the results, four clusters of ELFs were identified (A–D) and species of *Daldinia* sp., *Lasiodiplodia* sp. were found in all four clusters. *Daldinia* sp., *Lasiodiplodia* sp., *Neurospora* sp., *Rigidoporus vinctus*, *Cerrena* sp., *Endomelanconiopsis endophytica*, *Aspergillus hiratsukae*, *Nigrospora* sp., and *Xylaria psidii* were observed in group A; *Daldinia* sp., *Lasiodiplodia* sp., *Aspergillus* sp., *Diaporthe* sp., *Preussia* sp., *Hypoxylon* sp., *Xylariaceae* sp., *Neurospora* sp. and *Talaromyces* sp. were observed in group B; *Daldinia* sp., *Lasiodiplodia* sp., *Diaporthe* sp., *Endomelanconiopsis* sp., *Nigrospora* sp., *Schizophyllum* sp., *Neurospora* sp., and *Hypoxylon* sp. were observed in group C and *Daldinia* sp., *Lasiodiplodia* sp., *Preussia* sp., *Byssochlamys* sp. and *Endomelanconiopsis* sp. were observed in group D (Fig 9). The PCA analysis revealed that some of the ELFs belonged to the same species showed significant variations in their bioactivities. Similar scenario has been observed in Khan et al. (2016) and Chowdhary and Kaushik (2016) [37, 38]. No specific pattern or grouping of isolates based on metabolite production was found in PCA analysis. Pearson’s correlation analysis also indicated that there is no significant correlation among bioactivities.

In conclusion, endolichenic fungi have become a promising source to isolate novel bioactive compounds and there is a dearth of information on endolichenic fungi (ELF). The results obtained in this study will provide a starting point for discovering novel bioactive compounds from endolichenic fungi isolated from the lichens collected from mangrove and mangrove associated plants in Pullalam lagoon in Sri Lanka. In the present study, identification of 70 strains of ELF in lichens collected from mangroves were identified. The secondary metabolites produced by each ELF were assessed for antioxidant activity using DPPH, anti-diabetic activity using amylase inhibition and anti-obesity using lipase inhibition assays. The ethyl acetate extracts of *D*. *eschscholtzii* and *Sordaria* sp. showed better radical scavenging activity. Hence it is suggested that the extracts with low IC_50_ for the antioxidant assay and the fifteen fungal extracts with high antilipase activities might be of therapeutic interest with respect to the treatment of obesity and other non-communicable diseases.

Moreover, studies are required to determine the bioactive compounds responsible for the antioxidant and antilipase activities of each ELF extract.

## Supporting information

S1 File Sequence data of ITS-rDNA region of isolated ELF from lichens in Puttalam lagoon and their URLs in GenBank. (PDF)

S2 File Absorbance values obtain in DPPH assay (at 517nm) for all the ELF isolates used in the study. (PDF)

S3 File Absorbance values obtain in lipase inhibitory assay (at 405nm) for all the ELF isolates used in the study. (PDF)

S4 File Absorbance values obtain in amylase inhibitory assay (at 540nm) for all the ELF isolates used in the study. (PDF)

S5 File URLs of deposited sequence data in GenBank for endolichenic fungi collected from lichens in Puttalam lagoon. (DOCX)
