# Supplementary material for: Molecular phylogeny and bioprospecting of Endolichenic Fungi (ELF) inhabiting in the lichens collected from a mangrove ecosystem in Sri Lanka
Source: PLoS One. 2018 Aug 29;13(8):e0200711. doi: 10.1371/journal.pone.0200711 (PMC6114277; doi:10.1371/journal.pone.0200711)
Supplement: S1 File — (PDF) [file pone.0200711.s001.pdf]

S1 File. Sequences data and URLs of endolichenic fungi collected from lichens in Puttalam lagoon

Schizophyllum commune (AT_L1_E6)

https://www.ncbi.nlm.nih.gov/nuccore/MF773651.1

GACCTGCGGAAGGATCATTAACGAATCAAACAAGTTCATCTTGTTCTGATCCTGTGCACCTTATGTAGTC
CCAAAGCCTTCACGGGCGGCGGTTGACTACGTCTACCTCACACCTTAAAGTATGTTAACGAATGTAATCA
TGGTCTTGACAGACCCTAAAAAGTTAATACAACTTTCGACAACGGATCTCTTGGCTCTCGCATCGATGAA
GAACGCAGCGAAATGCGATAAGTAATGTGAATTGCAGAATTCAGTGAATCATCGAATCTTTGAACGCACC
TTGCGCCCTTTGGTATTCCGAGGGGCATGCCTGTTTGAGTGTCATTAAATACCATCAACCCTCTTTTGAC
TTCGGTCTCGAGAGTGGCTTGGAAGTGGAGGTCTGCTGGAGCCTAACGGAGCCAGCTCCTCTTAAATGTA
TTAGCGGATTTCCCTTGCGGGATCGCGTCTCCGATGTGATAATTTCTACGTCGTTGACCATCTCGGGGCT
GACCTAGTCAGTTTCAATAGGAGT

Schizophyllum commune (AT_L1_E7)

https://www.ncbi.nlm.nih.gov/nuccore/MF773657.1

GACCTGCGGAGGGATCATTGCTGGAACGCGCCCCAGGCGCACCCAGAAACCCTTTGTGAACTTATACCTT
TTGTTGCCTCGGCGCATGCTGGCCTCTAGTAGGCCCCTCACCCCGGTGAGGAGAAGGCACGCCGGCGGCC
AAGTTAACTCTTGTTTTTACACTGAAACTCTGAGAAAAAACACAAATGAATCAAAACTTTCAACAACGGA
TCTCTTGGTTCTGGCATCGATGAAGAACGCAGCGAAATGCGATAAGTAATGTGAATTGCAGAATTCAGTG
AATCATCGAATCTTTGAACGCACATTGCGCCCTCTGGTATTCCGGAGGGCATGCCTGTTCGAGCGTCATT
TCAACCCTCAAGCATTGCTTGGTGTTGGGGCACTGCTTTTAACGAAGCAGGCTCTGAAATCTAGTGGCGA
GCTCGCCAGGACCCCGAGCGCAGTAGTTAAACCCTCGCTCTGGAAGGCCCTGGCGGTGCCCTGCCGTTAA
ACCCCCAACTTCTGA

Diaporthe arengae (AT_L1_E1ST)

https://www.ncbi.nlm.nih.gov/nuccore/MF773656.1

GACCTGCGGAGGGATCATTGCTGGAACGCGCCCCAGGCGCACCCAGAAACCCTTTGTGAACTTATACCTT
TTGTTGCCTCGGCGCATGCTGGCCTCTAGTAGGCCCCTCACCCCGGTGAGGAGAAGGCACGCCGGCGGCC
AAGTTAACTCTTGTTTTTACACTGAAACTCTGAGAAAAAACACAAATGAATCAAAACTTTCAACAACGGA
TCTCTTGGTTCTGGCATCGATGAAGAACGCAGCGAAATGCGATAAGTAATGTGAATTGCAGAATTCAGTG
AATCATCGAATCTTTGAACGCACATTGCGCCCTCTGGTATTCCGGAGGGCATGCCTGTTCGAGCGTCATT
TCAACCCTCAAGCATTGCTTGGTGTTGGGGCACTGCTTTTAACGAAGCAGGCTCTGAAATCTAGTGGCGA
GCTCGCCAGGACCCCGAGCGCAGTAGTTAAACCCTCGCTCTGGAAGGCCCTGGCGGTGCCCTGCCGTTAA
ACCCCCAACTTCTGA

Aspergillus hiratsukae (AT_L2_E2)

https://www.ncbi.nlm.nih.gov/nuccore/MG593848.1

GGTGAACCTGCGGAAGGATCATTAACGAATCAAACAAGTTCATCTTGTTCTGATCCTGTGCACCTTATGT
AGTCCCAAAGCCTTCACGGGCGGCGGTTGACTACGTCTACCTCACACCTTAAAGTATGTTAACGAATGTA
ATCATGGTCTTGACAGACCCTAAAAAGTTAATACAACTTTCGACAACGGATCTCTTGGCTCTCGCATCGA
TGAAGAACGCAGCGAAATGCGATAAGTAATGTGAATTGCAGAATTCAGTGAATCATCGAATCTTTGAACG
CACCTTGCGCCCTTTGGTATTCCGAGGGGCATGCCTGTTTGAGTGTCATTAAATACCATCAACCCTCTTT
TGACTTCGGTCTCGAGAGTGGCTTGGAAGTGGAGGTCTGCTGGAGCCTAACGGAGCCAGCTCCTCTTAAA
TGTATTAGCGGATTTCCCTTGCGGGATCGCGTCTCCGATGTGATAATTTCTACGTCGTTGACCATCTCGG
GGCTGACCTAGTCAGTTTCAATAGGAGTCTGCTTCTAACCGTCTCTTGACTGAGACTAGCGACTTGTGCG
CTAA

Lasiodiplodia theobromae (AT_L4_E3)

https://www.ncbi.nlm.nih.gov/nuccore/KY992568.1

CGGCTCGACTCTCCCACCCTTTGTGAACGTACCTCTGTTGCTTTGGCGGCTCCGGCCGCCAAAGGACCTT
CAAACTCCAGTCAGTAAACGCAGACGTCTGATAAACAAGTTAATAAACTAAAACTTTCAACAACGGATCT
CTTGGTTCTGGCATCGATGAAGAACGCAGCGAAATGCGATAAGTAATGTGAATTGCAGAATTCAGTGAAT
CATCGAATCTTTGAACGCACATTGCGCCCCTTGGTATTCCGGGGGGCATGCCTGTTCGAGCGTCATTACA
ACCCTCAAGCTCTGCTTGGAATTGGGCACCGTCCTCACTGCGGACGCGCCTCAAAGACCTCGGCGGTGGC
TGTTCAGCCCTCAAGCGTAGTAGAATACACCTCGCTTTGGAGCGGTTGGCGTCGCCCGCCGGACGAACCT
TCTGAACTTTTCTCAAGGTTGACCTCGGATCAGGTAGGGATACCCGCTGAACTTAAGCATATCAA

Lasiodiplodia theobromae (AT_L5_E4)

https://www.ncbi.nlm.nih.gov/nuccore/KY992574.1

GCTCGACTCTCCCACCCTTTGTGAACGTACCTCTGTTGCTTTGGCGGCTCCGGCCGCCAAAGGACCTTCA
AACTCCAGTCAGTAAACGCAGACGTCTGATAAACAAGTTAATAAACTAAAACTTTCAACAACGGATCTCT
TGGTTCTGGCATCGATGAAGAACGCAGCGAAATGCGATAAGTAATGTGAATTGCAGAATTCAGTGAATCA
TCGAATCTTTGAACGCACATTGCGCCCCTTGGTATTCCGGGGGGCATGCCTGTTCGAGCGTCATTACAAC
CCTCAAGCTCTGCTTGGAATTGGGCACCGTCCTCACTGCGGACGCGCCTCAAAGACCTCGGCGGTGGCTG
TTCAGCCCTCAAGCGTAGTAGAATACACCTCGCTTTGGAGCGGTTGGCGTCGCCCGCCGGACGAACCTTC
TGAACTTTTCTCAAGGTTGACCTCGGATCAGGTAGGGATACCCGCTGAACTTAAGCATATCAAT

Sordaria sp. (AT_L3_E1)

https://www.ncbi.nlm.nih.gov/nuccore/KY992583.1

CCATCGCGAATCTTACCCGTACGGTTGCCTCGGCGCTGGCGGTCCGGAAGGCCCCTCGGAGCCCCCCGGA
CCCTCGGGTCTCCCGCTCGCGGGAGGCTGCCCGCCGGAGTGCCGAAACCAAACTCTTGATATTTTTATGT
CTCTCTGAGTAAACTTTTAAATAAGTCAAAACTTTCAACAACGGATCTCTTGGTTCTGGCATCGATGAAG
AACGCAGCGAAATGCGATAAGTAATGTGAATTGCAGAATTCAGTGAATCATCGAATCTTTGAACGCACAT
TGCGCTCGCCAGTATTCTGGCGAGCATGCCTGTTCGAGCGTCATTTCAACCATCAAGCTCTGCTTGCGTT
GGGGATCCGCGGCTGCCCGCGGTCCCTCAAAAACAGTG

Hypoxylon anthochroum (AT_L6_E5)

https://www.ncbi.nlm.nih.gov/nuccore/KY992584.1

CCCTATGTGACTTACCACTGTTGCCTCGGCGCTGTGCCTGCGAGAGCAGGCCCGCCGGTGGACCACTAAA
CTCTGTTATACCTACTGTATCTCTGAATTTATAACTGAAATACGTTAAAACTTTCAACAACGGATCTCTT
GGTTCTGGCATCGATGAAGAACGCAGCGAAATGCGATAAGTAATGTGAATTGCAGAATTCAGTGAATCAT
CGAATCTTTGAACGCACATTGCGCCCATTAGTATTCTAGTGGGCATGCCTATTCGAGCGTCATTTCAACC
CTTAAGCCCCTGCTGCTTAGTGTTGGGAATCTGCGTTACGGCGCAGTTCCTTAAAGTGATTTGGCGGAGC
TAGTGCATACTCTAGGCGTAGTAAAATACCATTCTCGCTTTTGTAGTAGGCCTGGCGGCTTGCCGTAAAA
CCCCTATATTTCTAGTGGTTGACCTCGGATTAGGTAGGAATACCCGCTGAACTTAAGCATATC

Aspergillus hiratsukae (AT_L6_E10)

https://www.ncbi.nlm.nih.gov/nuccore/KY977718.1

CCCTATGTGACTTACCACTGTTGCCTCGGCGCTGTGCCTGCGAGAGCAGGCCCGCCGGTGGACCACTAAA
CTCTGTTATACCTACTGTATCTCTGAATTTATAACTGAAATACGTTAAAACTTTCAACAACGGATCTCTT
GGTTCTGGCATCGATGAAGAACGCAGCGAAATGCGATAAGTAATGTGAATTGCAGAATTCAGTGAATCAT
CGAATCTTTGAACGCACATTGCGCCCATTAGTATTCTAGTGGGCATGCCTATTCGAGCGTCATTTCAACC
CTTAAGCCCCTGCTGCTTAGTGTTGGGAATCTGCGTTACGGCGCAGTTCCTTAAAGTGATTTGGCGGAGC
TAGTGCATACTCTAGGCGTAGTAAAATACCATTCTCGCTTTTGTAGTAGGCCTGGCGGCTTGCCGTAAAA
CCCCTATATTTCTAGTGGTTGACCTCGGATTAGGTAGGAATACCCGCTGAACTTAAGCATATC

Nigrospora sphaerica (AT_L6_E12)

https://www.ncbi.nlm.nih.gov/nuccore/KY977719.1

AATAAGTCAAAACTTTCAACAACGGATCTCTTGGTTCTGGCATCGATGAAGAACGCAGCGAAATGCGATA
AGTAATGTGAATTGCAGAATTCAGTGAATCATCGAATCTTTGAACGCACATTGCGCCCATTAGTATTCTA
GTGGGCATGCCTGTTCGAGCGTCATTTCAACCCCTAAGCACAGCTTATTGTTGGGCGTCTACGTCTGTAG
TGCCTCAAAGACATTGGCGGAGCGGCAGCAGTCCTCTGAGCGTAGTAATTCTTTATCTCGCTTCTGTTAG
GCGCTGCCCCCCCGGCCGTAAAACCCCCAA

Xylaria feejeensis (ATII_L6_E1)

https://www.ncbi.nlm.nih.gov/nuccore/MG593846.1

CCCCATGTGAACATACCTAACGTTGCCTCGGCGGGTCGTACCTACCTTGTAGTGCACTTACCTGTAAGTG
CCTACCCGGTAGGCACGGGTAAGCCCGCCGGCGCCCCACGAAACTCTGTTTAATTACTGGATATCTGAAT
TATAACTAAATAAGTTAAAACTTTCAACAACGGATCTCTTGGTTCTGGCATCGATGAAGAACGCAGCGAA
ATGCGATAAGTAATGTGAATTGCAGAATTCAGTGAATCATCGAATCTTTGAACGCACATTGCGCCCATTA
GTATTCTAGTGGGCATGCCTGTTCGAGCGTCATTTCAACCCTTAAGCCTTCTGTTGCTTAGCGTTGGGGG
CCTACCGTATGGCGGTAGCCCCTTAAAATTAGTGGCGGAGTCGGTTCACACTCTAGACGTAGTAAATATT
ATCTCGCCTATTAGTTGGACCGGTCCCCTGCCGTAAAACCCCTTAATTTTTCAAGGTTGACCTCGGATCA
GGTAGGAATACCCGCTGAACTTAAGCATATCAATA

Preussia sp. (ATII_L6_E5)

https://www.ncbi.nlm.nih.gov/nuccore/KY992581

TGTCGTGATAGAACCCTTGCCTTTTGAGTACCGTCCGTTTCCTCGGCAGGCTCGCCTGCCAATGGGGACC
CCCAATAAACCCTTTTTATGTACCTGTATCAGTCTGACAAACAAACAAAAGTTAAAACTTTCAACAACGG
ATCTCTTGGTTCTGGCATCGATGAAGAACGCAGCGAAATGCGATAAGTAGTGTGAATTGCAGAATTCAGT
GAATCATCGAATCTTTGAACGCACATTGCGCCCTTTGGTATTCCTTAGGGCATGCCTGTTCGAGCGTCAT
TTGTAACCTCAAGCTCAGCTTGGTGTTGGGTG

Neurospora crassa (AT_L7_E1)

https://www.ncbi.nlm.nih.gov/nuccore/KY992579.1

CCATCGCGAATCTTACCCGTACGGTTGCCTCGGCGCTGGCGGTCCGGAAAGGCCTTCGCGCCCTCCCGGA
TCCTCGGGTCTCCCGCTCGCGGGAGGCTGCCCGCCGGAGTGCCGAAACTAAACTCTTGATATTTTATGTC
TCTCTGAGTAAACTTTTAAATAAGTCAAAACTTTCAACAACGGATCTCTTGGTTCTGGCATCGATGAAGA
ACGCAGCGAAATGCGATAAGTAATGTGAATTGCAGAATTCAGTGAATCATCGAATCTTTGAACGCACATT
GCGCTCGCCAGTATTCTGGCGAGCATGCCTGTTCGAGCGTCATTTCAACCATCAAGCTCTGCTTGCGTTG

GGGATCCGCGGCTGTCCGCGGTCCCTCAAA

Nigrospora sp. (AT_L8_E1)

https://www.ncbi.nlm.nih.gov/nuccore/KY992575.1

GCCTGCGGAAGGATCATTACCGAGTTTTCGGGCTTCGGCTCGACTCTCCCACCCTTTGTGAACGTACCTC
TGTTGCTTTGGCGGCTCCGGCCGCCAAAGGACCTCCAAACTCCAGTCAGTAAACGCAGACGTCTGATAAA
CAAGTTAATAAACTAAAACTTTCAACAACGGATCTCTTGGTTCTGGCATCGATGAAGAACGCAGCGAAAT
GCGATAAGTAATGTGAATTGCAGAATTCAGTGAATCATCGAATCTTTGAACGCACATTGCGCCCCTTGGT
ATTCCGGGGGGCATGCCTGTTCGAGCGTCATTACAACCCTCAAGCTCTGCTTGGAATTGGGCACCGTCCT
CACTGCGGACGCGCCTCAAAGACCTCGGCGGTGGCTGTTCAGCCCTCAAGCGTAGTAGAATACACCTCGC
TTTGGAGTGGTTGGCGTCGCCCGCCGGACGAACCTTCTGAACTTTTCTCAAGGTGACCTCGGATC

Daldinia eschscholtzii (AT_L8_E5)

https://www.ncbi.nlm.nih.gov/nuccore/KY977720.1

GACCTGCGGAGGGATCATTACTGAGTTATCTAAACTCCAACCCTATGTGAACTTACCGCCGTTGCCTCGG
CGGGCCGCGTTCGCCCTGTAGTTTACTACCTGGCGGCGCGCTACAGGCCCGCCGGTGGACTGCTAAACTC
TGTTATACATACGTATCTCTGAATGCTTCAACTTAATAAGTTAAAACTTTCAACAACGGATCTCTTGGTT
CTGGCATCGATGAAGAACGCAGCGAAATGCGATAAGTAATGTGAATTGCAGAATTCAGTGAATCATCGAA
TCTTTGAACGCACATTGCGCCCATTAGTATTCTAGTGGGCATGCCTGTTCGAGCGTCATTTCAACCCTTA
AGCCCCTGTTGCTTAGCGTTGGGAATCTAGGTCTCCAGGGCCTAGTTCCCCAAAGTCATCGGCGGAGTCG
GAGCGTACTCTCAGCGTAGTAATACCATTCTCGCTTTTGCAGTAGCCCCGGCGGCTTGCCGTAAAACCCC
TATATCTTTAGTGGTGA

Cerrena unicolor (AT_L8_E12)

https://www.ncbi.nlm.nih.gov/nuccore/KY977721.1

AACCTGCGGAAGGATCATTAATGAATTTTATGGCGGAATTGTAGCTGGCCCCAACCGGGCATGTGCACAT
TCTGTTCATTCCATTCTCATACACCTCTGTGCACTTTACATAGGTTTGGTATAGAAAAGGTCTTTATTGA
CTTTGGAAATACTGACCTATGCTTTTATAAACGCTTCAGTTTTAGAATGTCATCCGCGTATAACGCAATA
AATACAACTTTCAGCAACGGATCTCTTGGCTCTCGCATCGATGAAGAACGCAGCGAAATGCGATAAGTAA
TGTGAATTGCAGAATTCAGTGAATCATCGAATCTTTGAACGCATCTTGCGCCCTTTGGTATTCCGAAGGG
CATGCCTGTTTGAGTGTCATGGTATTCTCAATACCCCAAATCTTTGCGGATAAGGGTGTGTTGGACTTGG
AGGTTTTTGCAGGTAATGATTGTGTTACCAGCTCCTCTTAAATGCATTAGCAGAGATAATACTGCTACTC
TCCAGTGTGATAATTGTCTACACTGTTAGTAGTGCGGTATAACAAAATGTCTATGCTTCTAATCGTCTTC
GGACAACTTTTGACAATCTGACCTC

Daldinia eschscholtzii (AT_L9_E1)

https://www.ncbi.nlm.nih.gov/nuccore/MF773660.1

GGGGTGACCTGCGGAGGGATCATTACTGAGTTATCTAAACTCCAACCCTATGTGAACTTACCGCCGTTGC
CTCGGCGGGCCGCGTTCGCCCTGTAGTTTACTACCTGGCGGCGCGCTACAGGCCCGCCGGTGGACTGCTA
AACTCTGTTGTATATACGTATCTCTGAATGCTTCAACTTAATAAGTTAAAACTTTCAACAACGGATCTCT
TGGTTCTGGCATCGATGAAGAACGCAGCGAAATGCGATAAGTAATGTGAATTGCAGAATTCAGTGAATCA
TCGAATCTTTGAACGCACATTGCGCCCATTAGTATTCTAGTGGGCATGCCTGTTCGAGCGTCATTTCAAC
CCTTAAGCCCCTGTTGCTTAGCGTTGGGAATCTAGGTCTCCAGGGCCTAGTTCCCCAAAGTCATCGGCGG
AGTCGGAGCGTACTCTCAGCGTAGTAATACCATTCTCGCTTTTGCAGTAGCCCCGGCGGCTTGCCGTAAA
ACCCCTATATCTTTAGTGGTTGACCT

Aspergillus hiratsukae (AT_L11_E3)

https://www.ncbi.nlm.nih.gov/nuccore/KY977724.1

TGACCTGCGGAAGGATCATTACCGAGTTTTCGGGCTCCGGCTCGACTCTCCCACCCTTTGTGAACGTACC
TCTGTTGCTTTGGCGGCTCCGGCCGCCAAAGGACCTTCAAACTCCAGTCAGTAAACGCAGACGTCTGATA
AACAAGTTAATAAACTAAAACTTTCAACAACGGATCTCTTGGTTCTGGCATCGATGAAGAACGCAGCGAA
ATGCGATAAGTAATGTGAATTGCAGAATTCAGTGAATCATCGAATCTTTGAACGCACATTGCGCCCCTTG
GTATTCCGGGGGGCATGCCTGTTCGAGCGTCATTACAACCCTCAAGCTCTGCTTGGAATTGGGCACCGTC
CTCACTGCGGACGCGCCTCAAAGACCTCGGCGGTGGCTGTTCAGCCCTCAAGCGTAGTAGAATACACCTC
GCTTTGGAGCGGTTGGCGTCGCCCGCCGGACGAACCTTCTGAACTTTTCTCAAGG

Endomelanconiopsis endophytica (AT_L11_E1)

https://www.ncbi.nlm.nih.gov/nuccore/KY977723.1

ACCTGCGGAAGGATCATTACCGAGTTCTAGGGGTCTTCGGACCTCTTCTCTCACACCCTATGTGTACCTA
CCTCTGTTGCTTTGGCGGGCCGCGGTCCTCCGCGGCCGGCCCCCTAACCGGGGCTGGCCAGCGCCCGCCA
GAGGACTACCAAACTCCAGTCAGTAAACGTAGCTGTCTGATCAAAAGTTTAATAAACTAAAACTTTCAAC
AACGGATCTCTTGGTTCTGGCATCGATGAAGAACGCAGCGAAATGCGATAAGTAATGTGAATTGCAGAAT
TCAGTGAATCATCGAATCTTTGAACGCACATTGCGCCCCTTGGTATTCCGAGGGGCATGCCTGTTCGAGC
GTCATTTCACCACTCAAGCTCTGCTTGGTATTGGGCGCCGTCCTTCACCGGACGCGCCTCAAAGACCTCG
GCGGTGGCGTCTTGCCTCAAGCGTAGTAGAAAACACCTCGCTTTGGAGGACGGGACGTTCGCTCGCCGGA
CGAACCTTCTGAATTTTCTCAAGGTGACCTCGGATCA

Aspergillus hiratsukae (AT_L12_E2)

https://www.ncbi.nlm.nih.gov/nuccore/KY977725.1

CCGGTAAGGGTGACCTGCGGAAGGATCATTAACCGAGTGAGGGGCCCTCCCGGGTCCCAACCTTCCCACA
CCGTGTCTATGCGTACCTTGTTGCTTCGGCGGGCCCCGCCGTTTCGACGGCCGCCGGGGAGGCCTTGCGC
CCCCGGGCCCGCGCCCGCCGAAGACCCCAACATGAACGCTGTTCTGAAAGTATGCAGTCTGAGTTGATTA
TCGTAATCAGTTAAAACTTTCAACAACGGATCTCTTGGTTCCGGCATCGATGAAGAACGCAGCGAAATGC
GATAAGTAATGTGAATTGCAGAATTCAGTGAATCATCGAGTCTTTGAACGCACATTGCGCCCCCTGGTAT
TCCGGGGGGCATGCCTGTCCGAGCGTCATTGCTGCCCTCAAGCACGGCTTGTGTGTTGGGCCCCCGTCCC
CCTCTCCCGGGGGACGGGCCCGAAAGGCAGCGGCGGCACCGCGTCCGGTCCTCGAGCGTATGGGGCTTTG
TCACC

Neurospora crassa (AT_L12_E4ST)

https://www.ncbi.nlm.nih.gov/nuccore/KY992573

CCATCGCGAATCTTACCCGTACGGTTGCCTCGGCGCTGGCGGTCCGGAAAGGCCTTCGCGCCCTCCCGGA
TCCTCGGGTCTCCCGCTCGCGGGAGGCTGCCCGCCGGAGTGCCGAAACTAAACTCTTGATATTTTATGTC
TCTCTGAGTAAACTTTTAAATAAGTCAAAACTTTCAACAACGGATCTCTTGGTTCTGGCATCGATGAAGA
ACGCAGCGAAATGCGATAAGTAATGTGAATTGCAGAATTCAGTGAATCATCGAATCTTTGAACGCACATT
GCGCTCGCCAGTATTCTGGCGAGCATGCCTGTTCGAGCGTCATTTCAACCATCAAGCTCTGCTTGCGTTG
GGGATCCGCGGCTGTCCGCGGTCCCTCAAAATCAGTGGCGGGCTCGCTAGTCACACCGAGCGTAGTAACT
CTACATCGCTATGGTCGTGCGGCGGGTTCTTGCCGTAAAACCCCCCATTTCTAAGGTTGACCTCGGATCA
GGTAGGAATACCCGCTGAACTTAAGCATAT

Xylaria psidii (AT_L13_E2)

https://www.ncbi.nlm.nih.gov/nuccore/MF773655.1

GCTCGACTCTCCCACCCTTTGTGAACGTACCTCTGTTGCTTTGGCGGCTCCGGCCGCCAAAGGACCTTCA
AACTCCAGTCAGTAAACGCAGACGTCTGATAAACAAGTTAATAAACTAAAACTTTCAACAACGGATCTCT
TGGTTCTGGCATCGATGAAGAACGCAGCGAAATGCGATAAGTAATGTGAATTGCAGAATTCAGTGAATCA
TCGAATCTTTGAACGCACATTGCGCCCCTTGGTATTCCGGGGGGCATGCCTGTTCGAGCGTCATTACAAC
CCTCAAGCTCTGCTTGGAATTGGGCACCGTCCTCACTGCGGACGCGCCTCAAAGACCTCGGCGGTGGCTG
TTCAGCCCTCAAGCGTAGTAGAATACACCTCGCTTTGGAGCGGTTGGCGTCGCCCGCCGGACGAACCTTC
TGAACTTTTCTCAAGGTTGACCTCGGATCAGGTAGGGATACCCGCTGAACTTAAGCATATCAATA

Daldinia eschscholtzii (NT_L1_E1)

https://www.ncbi.nlm.nih.gov/nuccore/KY992578.1

TCCACCCTATGTGAACTTACCGCCGTTGCCTCGGCGGGCCGCGTTCGCCCTGTAGTTTACTACCTGGCGG
CGCGCTACAGGCCCGCTGGTGGACTGCTAAACTCTGTTATATATACGTATCTCTGAATGCTTCAACTTAA
TAAGTTAAAACTTTCAACAACGGATCTCTTGGTTCTGGCATCGATGAAGAACGCAGCGAAATGCGATAAG
TAATGTGAATTGCAGAATTCAGTGAATCATCGAATCTTTGAACGCACATTGCGCCCATTAGTATTCTAGT
GGGCATGCCTGTTCGAGCGTCATTTCAACCCTTAAGCCCCTGTTGCTTAGCGTTGGGAATCTAGGTCTCC
AGGGCCTAGTTCCCCAAAGTCATCGGCGGAGTCGGAGCGTACTCTCAGCGTAGTAATACCATTCTCGCTT
TTGCAGTAGCCCCGGCGGCTTGCCGTAAAACCCCTATATCTTTAGTGGTTGACCTCGAATCAGGTAGGAA
TACCCGCTGAACTTAAGCATATC

Lasiodiplodia theobromae (NT_L1_E3)

https://www.ncbi.nlm.nih.gov/nuccore/KY977731.1

GACCTGCGGAAGGATCATTACCGAGTTTTCGGGCTCCGGCTCGACTCTCCCACCCTTTGTGAACGTACCT
CTGTTGCTTTGGCGGCTCCGGCCGCCAAAGGACCTTCAAACTCCAGTCAGTAAACGCAGACGTCTGATAA
ACAAGTTAATAAACTAAAACTTTCAACAACGGATCTCTTGGTTCTGGCATCGATGAAGAACGCAGCGAAA
TGCGATAAGTAATGTGAATTGCAGAATTCAGTGAATCATCGAATCTTTGAACGCACATTGCGCCCCTTGG
TATTCCGGGGGGCATGCCTGTTCGAGCGTCATTACAACCCTCAAGCTCTGCTTGGAATTGGGCACCGTCC
TCACTGCGGACGCGCCTCAAAGACCTCGGCGGTGGCTGTTCAGCCCTCAAGCGTAGTAGAATACACCTCG
CTTTGGAGCGGTTGGCGTCGCCCGCCGGACGAACCTTCTGAACTTTTCTCAAGGTGACCTCGGATCA

Daldinia eschscholtzii (NT_L2_E1)

https://www.ncbi.nlm.nih.gov/nuccore/KY992576.1

CCCTATGTGACTTACCGCCGTTGCCTCGGCGGGCCGCGTTCGCCCTGTAGTTTACTACCTGGCGGCGCGC
TACAGGCCCGCCGGTGGACTGCTAAACTCTGTTATATATACGTATCTCTGAATGCTTCAACTTAATAAGT
TAAAACTTTCAACAACGGATCTCTTGGTTCTGGCATCGATGAAGAACGCAGCGAAATGCGATAAGTAATG
TGAATTGCAGAATTCAGTGAATCATCGAATCTTTGAACGCACATTGCGCCCATTAGTATTCTAGTGGGCA
TGCCTGTTCGAGCGTCATTTCAACCCTTAAGCCCCTGTTGCTTAGCGTTGGGAATCTAGGTCTCCAGGGC
CTAGTTCCCCAAAGTCATCGGCGGAGTCGGAGCGTACTCTCAGCGTAGTAATACCATTCTCGCTTTTGCA
GTAGCCCCGGCGCCTTGCCGTAAAACCCCTATATCTTTAGTGGTTGACCTCGAATCAGGTAGGAATACCC
GCTGAACTTAAGCATATCA

Daldinia eschscholtzii (NT_L3_E1)

https://www.ncbi.nlm.nih.gov/nuccore/MF773669.1

GAACCTGCGGAGGGATCATTACTGAGTTATCTAAACTCCAACCCTATGTGAACTTACCGCCGTTGCCTCG
GCGGGCCGCGTTCGCCCTGTAGTTTACTACCTGGCGGCGCGCTACAGGCCCGCCGGTGGACTGCTAAACT
CTGTTATATATACGTATCTCTGAATGCTTCAACTTAATAAGTTAAAACTTTCAACAACGGATCTCTTGGT
TCTGGCATCGATGAAGAACGCAGCGAAATGCGATAAGTAATGTGAATTGCAGAATTCAGTGAATCATCGA
ATCTTTGAACGCACATTGCGCCCATTAGTATTCTAGTGGGCATGCCTGTTCGAGCGTCATTTCAACCCTT
AAGCCCCTGTTGCTTAGCGTTGGGAATCTAGGTCTCCAGGGCCTAGTTCCCCAAAGTCATCGGCGGAGTC
GGAGCGTACTCTCAGCGTAGTAATACCATTCTCGCTTTTGCAGTAGCCCCGGCGGCTTGCCGTAAAACCC
CTATATCTTTAGTGGTGACCTCGAATCA

Xylariaceae sp. (N_L1_E3)

https://www.ncbi.nlm.nih.gov/nuccore/MF773661.1

GGTGACCTGCGGAGGGATCATTACTGAGTTGTCTAAACTCCCAACCCTATGTGAACCTTACCGTCGTTGC
CTCGGCGGGTTGGGGAGCTACCCTGTAGTAGCCCCCCGTACGGCCCGCCGGTGGACTGTAAACTCTTGTT
TTCTGTATGGAATTCTGAATGCTTCAACTTAATAAGTTAAAACTTTCAACAACGGATCTCTTGGTTCTGG
CATCGATGAAGAACGCAGCGAAATGCGATAAGTAATGTGAATTGCAGAATTCAGTGAATCATCGAATCTT
TGAACGCACATTGCGCCCATTAGTATTCTAGTGGGCATGCCTATTCGAGCGTCATTTCAACCCTTAAGCC
CTAGTCGCTTAGCGTTGGGAGTCTGAGCCCTTCAGGGGGCTCAGTTCCTCAAAGTCATCGGCGGAGTCGG
ATCGTGCTCTGAGCGTAGTAATTTTCTCTCGCTTCTGAGGCCGTTCCGGTGACTGGCCGTAAAACCCCTA
TACTTCTAGTGGTGACC

Daldinia eschscholtzii (N_L2_E4)

https://www.ncbi.nlm.nih.gov/nuccore/MF773663.1

GAACCTGCGGAGGGATCATTACTGAGTTATCTAAACTCCAACCCTATGTGAACTTACCGCCGTTGCCTCG
GCGGGCCGCGTTCGCCCTGTAGTTTACTACCTGGCGGCGCGCTACAGGCCCGCCGGTGGACTGCTAAACT
CTGTTATACATACGTATCTCTGAATGCTTCAACTTAATAAGTTAAAACTTTCAACAACGGATCTCTTGGT
TCTGGCATCGATGAAGAACGCAGCGAAATGCGATAAGTAATGTGAATTGCAGAATTCAGTGAATCATCGA
ATCTTTGAACGCACATTGCGCCCATTAGTATTCTAGTGGGCATGCCTGTTCGAGCGTCATTTCAACCCTT
AAGCCCCTGTTGCTTAGCGTTGGGAATCTAGGTCTCCAGGGCCTAGTTCCCCAAAGTCATCGGCGGAGTC
GGAGCGTACTCTCAGCGTAGTAATACCATTCTCGCTTTTGCAGTAGCCCCGGCGGCTTGCCGTAAAACCC
CTATATCTTTAGTGGTGACCTCGAATCC

Xylaria castorea (N_L2_E7)

https://www.ncbi.nlm.nih.gov/nuccore/MF773662.1

GACCTGCGGAGGGATCATTAAAGAGTTCTATAACTCCCAAACTCATGTGAACATACCTTACGTTGCCTCG
GCAAGTCGCGCCTACCCAGTAGCGCCCTACCCTGTAGGGCCTACCCGGTAGACTCGGGTAAGCTTGCCGG
CGGCCTACGAAACTCTGTTTAATATTGAATTCTGAACGATAACCAAATAAAGTTAAAACTTTCAACAACG
GATCTCTTGGTTCTGGCATCGATGAAGAACGCAGCGAAATGCGATAAGTAATGTGAATTGCAGAATTCAG
TGAATCATCGAATCTTTGAACGCACATTGCGCCCATTAGTATTCTAGTGGGCATGCCTGTTCGAGCGTCA
TTTCAACCCTTAAGCCTTTGTTGCTTAGTGTTGGGAGCCTACGGTATTAGTAGCTCCTTAAAACTAGTGG
CGGAGTCGGTTCACACTCTAGACGTAGTAATTTTTATCTCGC

Diaporthe musigena (N_L4_E11)

https://www.ncbi.nlm.nih.gov/nuccore/KY977726.1

GACCTGCGGAGGGATCATTGCTGGAACGCGCCCCAGGCGCACCCAGAAACCCTTTGTGAACTTATACCTT
TTGTTGCCTCGGCGCATGCTGGCCTCTAGTAGGCCCCTCACCCCGGTGAGGAGAAGGCACGCCGGCGGCC
AAGTTAACTCTTGTTTTTACACTGAAACTCTGAGAAAAAACACAAATGAATCAAAACTTTCAACAACGGA
TCTCTTGGTTCTGGCATCGATGAAGAACGCAGCGAAATGCGATAAGTAATGTGAATTGCAGAATTCAGTG
AATCATCGAATCTTTGAACGCACATTGCGCCCTCTGGTATTCCGGAGGGCATGCCTGTTCGAGCGTCATT
TCAACCCTCAAGCATTGCTTGGTGTTGGGGCACTGCTTTTAACGAAGCAGGCCCTGAAATCTAGTGGCGA
GCTCGCCAGGACCCCGAGCGCAGTAGTTAAACCCTCGCTCTGGAAGGCCCTGGCGGTGCCCTGCCGTTAA
ACCCCCAACTTCTGAAA

Diaporthe arengae (N_L4_E23)

https://www.ncbi.nlm.nih.gov/nuccore/KY977727.1

GACCTGCGGAGGGATCATTGCTGGAACGCGCCCCAGGCGCACCCAGAAACCCTTTGTGAACTTATACCTT
TTGTTGCCTCGGCGCATGCTGGCCTCTAGTAGGCCCCTCACCCCGGTGAGGAGAAGGCACGCCGGCGGCC
AAGTTAACTCTTGTTTTTACACTGAAACTCTGAGAAAAAACACAAATGAATCAAAACTTTCAACAACGGA
TCTCTTGGTTCTGGCATCGATGAAGAACGCAGCGAAATGCGATAAGTAATGTGAATTGCAGAATTCAGTG
AATCATCGAATCTTTGAACGCACATTGCGCCCTCTGGTATTCCGGAGGGCATGCCTGTTCGAGCGTCATT
TCAACCCTCAAGCATTGCTTGGTGTTGGGGCACTGCTTTTAACGAAGCAGGCCCTGAAATCTAGTGGCGA
GCTCGCCAGGACCCCGAGCGCAGTAGTTAAACCCTCGCTCTGGAAGGCCCTGGCGGTGCCCTGCCGTTAA
ACCCCCAACTTCTGAAAA

Daldinia sp. (N_L5_E2)

https://www.ncbi.nlm.nih.gov/nuccore/MF773665.1

CCTGCGGAGGGATCATTACTGAGTTATCTAAACTCCAACCCTATGTGAACTTACCGCCGTTGCCTCGGCG
GGCCGCGTTCGCCCTGTAGTTTACTACCTGGCGGCGCGCTACAGGCCCGCCGGTGGACTGCTAAACTCTG
TTATATATACGTATCTCTGAATGCTTCAACTTAATAAGTTAAAACTTTCAACAACGGATCTCTTGGTTCT
GGCATCGATGAAGAACGCAGCGAAATGCGATAAGTAATGTGAATTGCAGAATTCAGTGAATCATCGAATC
TTTGAACGCACATTGCGCCCATTAGTATTCTAGTGGGCATGCCTGTTCGAGCGTCATTTCAACCCTTAAG
CCCCTGTTGCTTAGCGTTGGGAATCTAGGTCTCCAGGGCCTAGTTCCCCAAAGTCATCGGCGGAGTCGGA
GCGTACTCTCAGCGTAGTAATACCATTCTCGCTTTTGCAGTAGCCCCGGCGGCTTGCCGTAAAACCCCTA
TATC

Preussia tenerifae (N_L6_E1)

https://www.ncbi.nlm.nih.gov/nuccore/KY992582.1

CCCACAAACCATCGCGAATCTTACCCGTACGGTTGCCTCGGCGCTGGCGGTCCGGAAGGCCCCTCGGAGC
CCCCCGGACCCTCGGGTCTCCCGCTCGCGGGAGGCTGCCCGCCGGAGTGCCGAAACCAAACTCTTGATAT
TTTTATGTCTCTCTGAGTAAACTTTTAAATAAGTCAAAACTTTCAACAACGGATCTCTTGGTTCTGGCAT
CGATGAAGAACGCAGCGAAATGCGATAAGTAATGTGAATTGCAGAATTCAGTGAATCATCGAATCTTTGA
ACGCACATTGCGCTCGCCAGTATTCTGGCGAGCATGCCTGTTCGAGCGTCATTTCAACCATCAAGCTCTG
CTTGCGTTGGGGA

Nigrospora sp. (N_L7_E3)

https://www.ncbi.nlm.nih.gov/nuccore/KY992566.1

CCATGTGACATATCTCTTTGTTGCCTCGGCGCAAGCTACCCGGGACCTCGCGCCCCGGGCGGCCCGCCGG
CGGACAAACCAAACTCTGTTATCTTCGTTGATTATCTGAGTGTCTTATTTAATAAGTCAAAACTTTCAAC
AACGGATCTCTTGGTTCTGGCATCGATGAAGAACGCAGCGAAATGCGATAAGTAATGTGAATTGCAGAAT
TCAGTGAATCATCGAATCTTTGAACGCACATTGCGCCCATTAGTATTCTAGTGGGCATGCCTGTTCGAGC
GTCATTTCAACCCCTAAGCACAGCTTATTGTTGGGCGTCTACGTCTGTAGTGCCTCAAAGACATTGGCGG
AGCGGCAGCAGTCCTCTGAGCGTAGTAATTCTTTATCTCGCTTCTGTTAGGCGCTGCCCCCCCGGCCGTA
AAACCCCCAATTTTTTCTGGTTGACCTCGGATCAGGTAGGAATACCCGCTGAACTTAAGCATATCAATA

Rigidoporus vinctus (N_L7_E6)

https://www.ncbi.nlm.nih.gov/nuccore/KY992567.1

CATGTGCACATTCTGTTCATTCCATTCTCATACACCTCTGTGCACTTTACATAGGTTTGGTATAGAAAAG
GTCTTTATTGACTTTGGAAATACTGACCTATGCTTTTATAAACGCTTCAGTTTTAGAATGTCATCCGCGT
ATAACGCAATAAATACAACTTTCAGCAACGGATCTCTTGGCTCTCGCATCGATGAAGAACGCAGCGAAAT
GCGATAAGTAATGTGAATTGCAGAATTCAGTGAATCATCGAATCTTTGAACGCATCTTGCGCCCTTTGGT
ATTCCGAAGGGCATGCCTGTTTGAGTGTCATGGTATTCTCAATACCCCAAATCTTTGCGGATAAGGGTGT
GTTGGACTTGGAGGTTTTTGCAGGTAATGATTGTGTTACCAGCTCCTCTTAAATGCATTAGCAGAGATAA
TACTGCTACTCTCCAGTG

Lasiodiplodia theobromae (N_L8_E2)

https://www.ncbi.nlm.nih.gov/nuccore/KY992571.1

GCTCGACTCTCCCACCCTTTGTGAACGTACCTCTGTTGCTTTGGCGGCTCCGGCCGCCAAAGGACCTTCA
AACTCCAGTCAGTAAACGCAGACGTCTGATAAACAAGTTAATAAACTAAAACTTTCAACAACGGATCTCT
TGGTTCTGGCATCGATGAAGAACGCAGCGAAATGCGATAAGTAATGTGAATTGCAGAATTCAGTGAATCA
TCGAATCTTTGAACGCACATTGCGCCCCTTGGTATTCCGGGGGGCATGCCTGTTCGAGCGTCATTACAAC
CCTCAAGCTCTGCTTGGAATTGGGCACCGTCCTCACTGCGGACGCGCCTCAAAGACCTCGGCGGTGGCTG
TTCAGCCCTCAAGCGTAGTAGAATACACCTCGCTTTGGAGCGGTTGGCGTCGCCCGCCGGACGAACCTTC
TGAACTTTTCTCAAGGTTGACCTCGGATCAGGTAGGGATACCCGCTGAACTTAAGCATATCAATA

Lasiodiplodia pseudotheobromae (N_L8_E1)

https://www.ncbi.nlm.nih.gov/nuccore/KY977728.1

GCCTGCGGAAGGATCATTACCGAGTTTTCGGGCTTCGGCTCGACTCTCCCACCCTTTGTGAACGTACCTC
TGTTGCTTTGGCGGCTCCGGCCGCCAAAGGACCTCCAAACTCCAGTCAGTAAACGCAGACGTCTGATAAA
CAAGTTAATAAACTAAAACTTTCAACAACGGATCTCTTGGTTCTGGCATCGATGAAGAACGCAGCGAAAT
GCGATAAGTAATGTGAATTGCAGAATTCAGTGAATCATCGAATCTTTGAACGCACATTGCGCCCCTTGGT
ATTCCGGGGGGCATGCCTGTTCGAGCGTCATTACAACCCTCAAGCTCTGCTTGGAATTGGGCACCGTCCT
CACTGCGGACGCGCCTCAAAGACCTCGGCGGTGGCTGTTCAGCCCTCAAGCGTAGTAGAATACACCTCGC
TTTGGAGTGGTTGGCGTCGCCCGCCGGACGAACCTTCTGAACTTTTCTCAAGGTGACCTCGGATC

Lasiodiplodia theobromae (N_L9_E1)

https://www.ncbi.nlm.nih.gov/nuccore/KY992570.1

TCGACTCTCCCACCCTTTGTGAACGTACCTCTGTTGCTTTGGCGGCTCCGGCCGCCAAAGGACCTTCAAA
CTCCAGTCAGTAAACGCAGACGTCTGATAAACAAGTTAATAAACTAAAACTTTCAACAACGGATCTCTTG
GTTCTGGCATCGATGAAGAACGCAGCGAAATGCGATAAGTAATGTGAATTGCAGAATTCAGTGAATCATC
GAATCTTTGAACGCACATTGCGCCCCTTGGTATTCCGGGGGGCATGCCTGTTCGAGCGTCATTACAACCC
TCAAGCTCTGCTTGGAATTGGGCACCGTCCTCACTGCGGACGCGCCTCAAAGACCTCGGCGGTGGCTGTT
CAGCCCTCAAGCGTAGTAGAATACACCTCGCTTTGGAGCGGTTGGCGTCGCCCGCCGGACGAACCTTCTG
AACTTTTCTCAAGGTTGACCTCGGATCAGGTAGGGATACCCGCTGAACTTAAGCATATCAATAAGCGGAG
G

Daldinia eschscholtzii (N_L9_E4)

https://www.ncbi.nlm.nih.gov/nuccore/KY977729.1

GAACCTGCGGAGGGATCATTACTGAGTTATCTAAACTCCAACCCTATGTGAACTTACCGCCGTTGCCTCG
GCGGGCCGCGTTCGCCCTGTAGTTTACTACCTGGCGGCGCGCTACAGGCCCGCCGGTGGACTGCTAAACT
CTGTTATATATACGTATCTCTGAATGCTTCAACTTAATAAGTTAAAACTTTCAACAACGGATCTCTTGGT
TCTGGCATCGATGAAGAACGCAGCGAAATGCGATAAGTAATGTGAATTGCAGAATTCAGTGAATCATCGA
ATCTTTGAACGCACATTGCGCCCATTAGTATTCTAGTGGGCATGCCTGTTCGAGCGTCATTTCAACCCTT
AAGCCCCTGTTGCTTAGCGTTGGGAATCTAGGTCTCCAGGGCCTAGTTCCCCAAAGTCATCGGCGGAGTC
GGAGCGTACTCTCAGCGTAGTAATACCATTCTCGCTTTTGCAGTAGCCCCGGCGGCTTGCCGTAAAACCC
CTATATCTTTAGTGGTG

Byssochlamys spectabilis (N_L10_E4ST)

https://www.ncbi.nlm.nih.gov/nuccore/KY977730

TTCCGTAGGTGAACCTGCGGAAGGATCATTACCGAGTGAGGGTCCCTCGGGGCCCAACCTCCCATCCGTG
TTGTCCTGACACCTGTTGCTTCGGCGGGCCCGCCGTGGTTCACGCCCCGGCCGCCGGGGGGTTCACGCCC
CCGGGCCCGCGCCCGCCGAAGACCCCTGGAACGCTGCCTGGAAGGTTGCCGTATGAGTATACAATCAATC
AATTAAAACTTTCAACAACGGATCTCTTGGTTCCGGCATCGATGAAGAACGCAGCGAAATGCGATAAGTA
ATGTGAATTGCAGAATTCCGTGAATCATAGAATCTTTGAACGCACATTGCGCCCCCTGGCATTCCGGGGG
GCATGCCTGTCCGAGCGTCATTGCTAACCCTCCAGCCCGGCTGGTGAGTTGGGCCGCCGTCCCCCCCCCC
CGGGGGACGGGCCCGAAAGGCAGCGGCGGCGTCGCGTCCGGTCCTCGAGCGTATGGGGCTTTGTCACACG
CTTCAGTAGAACCGGCCGGCTTGCTGGCCACACGACCTTCACGGTCACCTATATTTCTCTTAGGTGACC

Chaetomium sp. (N_L10_E1)

https://www.ncbi.nlm.nih.gov/nuccore/MF773667.1

GAACCTGCGGAGGGATCATTACTGAGTTATCTAAACTCCAACCCTATGTGAACTTACCGCCGTTGCCTCG
GCGGGCCGCGTTCGCCCTGTAGTTTACTACCTGGCGGCGCGCTACAGGCCCGCCGGTGGACTGCTAAACT
CTGTTATATATACGTATCTCTGAATGCTTCAACTTAATAAGTTAAAACTTTCAACAACGGATCTCTTGGT
TCTGGCATCGATGAAGAACGCAGCGAAATGCGATAAGTAATGTGAATTGCAGAATTCAGTGAATCATCGA
ATCTTTGAACGCACATTGCGCCCATTAGTATTCTAGTGGGCATGCCTGTTCGAGCGTCATTTCAACCCTT
AAGCCCCTGTTGCTTAGCGTTGGGAATCTAGGTCTCCAGGGCCTAGTTCCCCAAAGTCATCGGCGGAGTC
GGAGCGTACTCTCAGCGTAGTAATACCATTCTCGCTTTTGCAGTAGCCCCGGCGGCTTGCCGTAAAACCC
CTATATCTTTAGTGGTG

Daldinia eschscholtzii (2_23)

https://www.ncbi.nlm.nih.gov/nuccore/MF773682.1

```
CCTGCGGAGGGATCATTACTGAGTTATCTAAACTCCAACCCTATGTGAACTTACCGCCGTTGCCTCGGCG
GGCCGCGTTCGCCCTGTAGTTTACTACCTGGCGGCGCGCTACAGGCCCGCCGGTGGACTGCTAAACTCTG
TTATATATACGTATCTCTGAATGCTTCAACTTAATAAGTTAAAACTTTCAACAACGGATCTCTTGGTTCT
GGCATCGATGAAGAACGCAGCGAAATGCGATAAGTAATGTGAATTGCAGAATTCAGTGAATCATCGAATC
TTTGAACGCACATTGCGCCCATTAGTATTCTAGTGGGCATGCCTGTTCGAGCGTCATTTCAACCCTTAAG
CCCCTGTTGCTTAGCGTTGGGAATCTAGGTCTCCAGGGCCTAGTTCCCCAAAGTCATCGGCGGAGTCGGA
GCGTACTCTCAGCGTAGTAATACCATTCTCGCTTTTGCAGTAGCCCCGGCGGCTTGCCGTAAAACCCCTA
TATCTTTAGTGGTGACCTCGAATCAG
```

Hypoxylon anthochroum (2_7)

https://www.ncbi.nlm.nih.gov/nuccore/KY985428

```
ACCTGCGGAGGGATCATTACTGAGTTCTAAACTCCAACCCTATGTGAACTTACCACTGTTGCCTCGGCGC
TGTGCCTGCGAGAGCAGGCCCGCCGGTGGACCACTAAACTCTGTTATACCTACTGTATCTCTGAATTTAT
AACTGAAATACGTTAAAACTTTCAACAACGGATCTCTTGGTTCTGGCATCGATGAAGAACGCAGCGAAAT
GCGATAAGTAATGTGAATTGCAGAATTCAGTGAATCATCGAATCTTTGAACGCACATTGCGCCCATTAGT
ATTCTAGTGGGCATGCCTATTCGAGCGTCATTTCAACCCTTAAGCCCCTGTTGCTTAGTGTTGGGAATCT
GCGTTACGGCGCAGTTCCTTAAAGTGATTTGGCGGAGCTAGTGCATACTCTAGGCGTAGTAAATACCATT
CTCGCTTTTGTAGTAGGCCTGGCGGCTTGCCGTAAAACCCCTATACTTCTAGTGGTTGACCTCGGA
```

Daldinia eschscholtzii (4_34)

https://www.ncbi.nlm.nih.gov/nuccore/MF029744.1

```
GGCTCGACTCTCCCACCCTTTGTGAACACACCTCTGTTGCCTCGGCGGCCGCTCCGGCCGCCGAAGGACC
TTCAAACTCCAGTCAGTAAACGCAGACGTCTGATAAACAAGTCAATAAACTAAAACTTTCAACAACGGAT
CTCTTGGTTCTGGCATCGATGAAGAACGCAGCGAAATGCGATAAGTAATGTGAATTGCAGAATTCAGTGA
ATCATCGAATCTTTGAACGCACATTGCGCCCCTTGGTATTCCGAGGGGCATGCCTGTTCGAGCGTCATTA
CAACCCTCAAGCTCTGCTTGGTATTGGGCTCCGTCCTCACTGCGGACGCGCCTCGAAGACCTCGGCGGTG
GCTGTTCGGCCCTCAAGCGTAGTAGAATACACCTCGCTTTGGAGCGGTCGGCGTCGCCCGCCGGACGAAC
CTTCTGAACTTTTCTCAAGGTGACCTCGGAT
```

Lasiodiplodia crassispora (4_22)

https://www.ncbi.nlm.nih.gov/nuccore/MF029743.1

```
CGTTGCCTCGGCGGGCCGCGTTCGCCCTGTAGTTTACTACCTGGCGGCGCGCTACAGGCCCGCCGGTGGA
CTGCTAAACTCTGTTATATATACGTATCTCTGAATGCTTCAACTTAATAAGTTAAAACTTTCAACAACGG
ATCTCTTGGTTCTGGCATCGATGAAGAACGCAGCGAAATGCGATAAGTAATGTGAATTGCAGAATTCAGT
GAATCATCGAATCTTTGAACGCACATTGCGCCCATTAGTATTCTAGTGGGCATGCCTGTTCGAGCGTCAT
TTCAACCCTTAAGCCCCTGTTGCTTAGCGTTGGGAATCTAGGTCTCCAGGGCCTAGTTCCCCAAAGTCAT
CGGCGGAGTCGGAGCGTACTCTCAGCGTAGTAATACCATTCTCGCTTTTGCAGTAGCCCCGGCGGCTTGC
CGTAAAAC
```

Daldinia eschscholtzii (5_30)

https://www.ncbi.nlm.nih.gov/nuccore/MF029745.1

CTTCGGGCGGCCCAGGGCCTGCCCCCGGGACCGCGCCCGCCGGAGACCCCAATGGAACACTGTCTGAAAG
CGTGCAGTCTGAGTCGATTGATACCAATCAGTCAAAACTTTCAACAATGGATCTCTTGGTTCCGGCATCG
ATGAAGAACGGAGCGAAATGCGATAACTAATGTGAATTGCAGAATTCAGTGAATCATCGAGTCTTTGAAC
GCACATTGCGCCCCCTGGTATTCCGGGGGGCATGCCTGTCCGAGCGTCATTTCTCCCCTCCAGCCCCGCT
GGTTGTTGGGCCGCGCCCCCCCGGGGGCGGGCCTCGAGAGAAACGGCGGCACCGTCCGGTCCTCGAGCGT
ATGGGGCTCTGTCACCCGCTCTATGGGCCCGGCCGGGGCTTGCCT

Hypoxylon anthochroum (5_12)

https://www.ncbi.nlm.nih.gov/nuccore/MF773672.1

ACCTGCGGAGGGATCATTACTGAGTTATCTAAACTCCAACCCTATGTGAACTTACCGCCGTTGCCTCGGC
GGGCCGCGTTCGCCCTGTAGTTTACTACCTGGCGGCGCGCTACAGGCCCGCCGGTGGACTGCTAAACTCT
GTTATATATACGTATCTCTGAATGCTTCAACTTAATAAGTTAAAACTTTCAACAACGGATCTCTTGGTTC
TGGCATCGATGAAGAACGCAGCGAAATGCGATAAGTAATGTGAATTGCAGAATTCAGTGAATCATCGAAT
CTTTGAACGCACATTGCGCCCATTAGTATTCTAGTGGGCATGCCTGTTCGAGCGTCATTTCAACCCTTAA
GCCCCTGTTGCTTAGCGTTGGGAATCTAGGTCTCCAGGGCCTAGTTCCCCAAAGTCATCGGCGGAGTCGG
AGCGTACTCTCAGCGTAGTAATACCATTCTCGCTTTTGCAGTAGCCCCGGCGGCTTGCCGTAAAACCCCT
ATATCTTTAGTGGTGACCTCGAATCAG

Xylariaceae sp. (6_12)

https://www.ncbi.nlm.nih.gov/nuccore/MF029748.1

CTGCGGAGGGATCATTACTGAGTTCTAAACTCCAACCCTATGTGAACTTACCACTGTTGCCTCGGCGCTG
TGCCTGCGAGAGCAGGCCCGCCGGTGGACCACTAAACTCTGCTATACCTACTGTATCTCTGAATTTATAA
CTGAAATACGTTAAAACTTTCAACAACGGATCTCTTGGTTCTGGCATCGATGAAGAACGCAGCGAAATGC
GATAAGTAATGTGAATTGCAGAATTCAGTGAATCATCGAATCTTTGAACGCACATTGCGCCCATTAGTAT
TCTAGTGGGCATGCCTATTCGAGCGTCATTTCAACCCTTAAGCCCCTGTTGCTTAGTGTTGGGAATCTGC
GTTACGGCGCAGTTCCTTAAAGTGATTTGGCGGAGCTAGTGCATACTCTAGGCGTAGTAAAATACCATTC
TCGCTTTTGTAGTAGGCCTGGCGGCTTGCCGTAAAACCCCTATATTTCTAGTGGTGACCTCGGATA

Schizophyllum commune (6_17)

https://www.ncbi.nlm.nih.gov/nuccore/MF773673.1

CCTGCGGAGGGATCATTACTGAGTTGTCTAAACTCCCAACCCTATGTGAACCTTACCGTCGTTGCCTCGG
CGGGTTGGGGAGCTACCCTGTAGTAGCCCCCCGTACGGCCCGCCGGTGGACTGTAAACTCTTGTTTTCTG
TATGGAATTCTGAATGCTTCAACTTAATAAGTTAAAACTTTCAACAACGGATCTCTTGGTTCTGGCATCG
ATGAAGAACGCAGCGAAATGCGATAAGTAATGTGAATTGCAGAATTCAGTGAATCATCGAATCTTTGAAC
GCACATTGCGCCCATTAGTATTCTAGTGGGCATGCCTATTCGAGCGTCATTTCAACCCTTAAGCCCTAGT
CGCTTAGCGTTGGGAGTCTGAGCCCTTCAGGGGGCTCAGTTCCTCAAAGTCATCGGCGGAGTCGGATCGT
GCTCTGAGCGTAGTAATTTTCTCTCGCTTCTGAGGCCGTTCCGGTGACTGGCCGTAAAACCCC

Daldinia eschscholtzii isolate 7_2

https://www.ncbi.nlm.nih.gov/nuccore/MF773674.1

CCTGCGGAAGGATCATTAACGAATCAAACAAGTTCATCTTGTTCTGATCCTGTGCACCTTATGTAGTCCC
AAAGCCTTCACGGGCGGCGGTTGACTACGTCTACCTCACACCTTAAAGTATGTTAACGAATGTAATCATG
GTCTTGACAGACCCTAAAAAGTTAATACAACTTTCGACAACGGATCTCTTGGCTCTCGCATCGATGAAGA
ACGCAGCGAAATGCGATAAGTAATGTGAATTGCAGAATTCAGTGAATCATCGAATCTTTGAACGCACCTT
GCGCCCTTTGGTATTCCGAGGGGCATGCCTGTTTGAGTGTCATTAAATACCATCAACCCTCTTTTGACTT
CGGTCTCGAGAGTGGCTTGGAAGTGGAGGTCTGCTGGAGCCTAACGGAGCCAGCTCCTCTTAAATGTATT
AGCGGATTTCCCTTGCGGGATCGCGTCTCCGATGTGATAATTTCTACGTCGTTGACCATCTCGGGGCTGA
CCTAGTCAGTTTCAATAGGAGTCTGCTTCCAACCGTCTCTTGACCGAGACTAGCGACTTGTGCGCTAACT
TTTGACTTGAC

Endomelanconiopsis sp. (8_8)

https://www.ncbi.nlm.nih.gov/nuccore/MF029751.1

CGGGGCTGGCCAGCGCCCGCCAGAGGACTACCAAACTCCAGTCAGTAAACGTAGCTGTCTGATCAAAAGT
TTAATAAACTAAAACTTTCAACAACGGATCTCTTGGTTCTGGCATCGATGAAGAACGCAGCGAAATGCGA
TAAGTAATGTGAATTGCAGAATTCAGTGAATCATCGAATCTTTGAACGCACATTGCGCCCCTTGGTATTC
CGAGGGGCATGCCTGTTCGAGCGTCATTTCACCACTCAAGCTCTGCTTGGTATTGGGCGCCGTCCTTCAC
CGGACGCGCCTCAAAGACCTCGGCGGTGGCGTCTTGCCTCAAGCGTAGTAGAAAACACCTCGCTTTGGAG
GACGGGACGTTCGCTCGCCGGACGAAC

Endomelanconiopsis endophytica (7_26)

https://www.ncbi.nlm.nih.gov/nuccore/MF029750.1

TGTGTACCTACCTCTGTTGCTTTGGCGGGCCGCGGTCCTCCGCGGCCGGCCCCCTAACCGGGGCTGGCCA
GCGCCCGCCAGAGGACTACCAAACTCCAGTCAGTAAACGTAGCTGTCTGATCAAAAGTTTAATAAACTAA
AACTTTCAACAACGGATCTCTTGGTTCTGGCATCGATGAAGAACGCAGCGAAATGCGATAAGTAATGTGA
ATTGCAGAATTCAGTGAATCATCGAATCTTTGAACGCACATTGCGCCCCTTGGTATTCCGAGGGGCATGC
CTGTTCGAGCGTCATTTCACCACTCAAGCTCTGCTTGGTATTGGGCGCCGTCCTTCACCGGACGCGCCTC
AAAGACCTCGGCGGTGGCGTCTTGCCTCAAGCGTAGTAGAAAACACCTCGCTTTGGAGGACGGGACGTTC
GCTCGCCGGACGAACCTTCTGAATTTTCTCAAGGTGACC

Aspergillus aculeatus (7_18)

https://www.ncbi.nlm.nih.gov/nuccore/MF773675.1

ACCTGCGGAAGGATCATTACCGAGTGCTGGGTCCTTCGGGGCCCAACCTCCCACCCGTGCTTACCGTACC
CTGTTGCTTCGGCGGGCCCGCCTTCGGGCGGCCCGGGGCCTGCCCCCGGGACCGCGCCCGCCGGAGACCC
CAATGGAACACTGTCTGAAAGCGTGCAGTCTGAGTCGATTGATACCAATCAGTCAAAACTTTCAACAATG
GATCTCTTGGTTCCGGCATCGATGAAGAACGCAGCGAAATGCGATAACTAATGTGAATTGCAGAATTCAG
TGAATCATCGAGTCTTTGAACGCACATTGCGCCCCCTGGTATTCCGGGGGGCATGCCTGTCCGAGCGTCA
TTTCTCCCCTCCAGCCCCGCTGGTTGTTGGGCCGCGCCCCCCCGGGGGCGGGCCTCGAGAGAAACGGCGG
CACCGTCCGGTCCTCGAGCGTATGGGGCTCTGTCACCCGCTCTATGGGCCCGGCCGGGGCTTGCCTCGAC
CCCCAATCTTCTCAGATGACC

Talaromyces pinophilus (7_19)

https://www.ncbi.nlm.nih.gov/nuccore/MF773676

CTGCGGAAGGATCATTACCGAGTGCGGGCCCTCGCGGCCCAACCTCCCACCCTTGTCTCTATACACCTGT
TGCTTTGGCGGGCCCACCGGGGCCACCTGGTCGCCGGGGGACGCACGTCCCCGGGCCCGCGCCCGCCGAA
GCGCTCTGTGAACCCTGATGAAGATGGGCTGTCTGAGTATTATGAAAATTGTCAAAACTTTCAACAATGG
ATCTCTTGGTTCCGGCATCGATGAAGAACGCAGCGAAATGCGATAAGTAATGTGAATTGCAGAATTCCGT
GAATCATCGAATCTTTGAACGCACATTGCGCCCCCTGGCATTCCGGGGGGCATGCCTGTCCGAGCGTCAT
TTCTGCCCTCAAGCACGGCTTGTGTGTTGGGTGTGGTCCCCCCGGGGACCTGCCCGAAAGGCAGCGGCGA
CGTCCGTCTGGTCCTCGAGCGTATGGGGCTTTGTCACTCGCTCGGGAAGGACCTGCGGGGGTTGGTCACC
ACCATATTTTACCACGGTTGACCTCGGATCACG

Phomopsis sp. (8_19)

https://www.ncbi.nlm.nih.gov/nuccore/MF773677.1

CCTGCGGAGGGATCATTGCTGGAACGCGCCCCAGGCGCACCCAGAAACCCTTTGTGAACTTATACCTTAC
TGTTGCCTCGGCGTATGCTGGCCCCTAGGGGTCCCTCTTGTCTCAAGAGGAGCAGGCACGCCGGCGGCCA
AGTCAACTCTTGTTTTTACACTGAAACTCTGAGAAAAAAACAAAAATGAATCAAAACTTTCAACAACGGA
TCTCTTGGTTCTGGCATCGATGAAGAACGCAGCGAAATGCGATAAGTAATGTGAATTGCAGAATTCAGTG
AATCATCGAATCTTTGAACGCACATTGCGCCCTCCGGTATTCCGGAGGGCATGCCTGTTCGAGCGTCATT
TCAACCCTCAAGCCTGGCTTGGTGTTGGGGCACTGCTTCTCTCGCGGGAAGCAGGCCCTCAAATCTAGTG
GCGAGCTCGCCAGGACCCCGAGCGTAGTAGTTAAACCCTCGCTTTGGAAGGCCCGGCGGTGCCCTGCCGT
TAAACCCCC

Cerrena sp. (9_10)

https://www.ncbi.nlm.nih.gov/nuccore/MF773678.1

CATTAATGAATTTTATGGCGGAATTGTAGCTGGCCCCAACCGGGCATGTGCACATTCTGTTCATTCCATT
CTCATACACCTCTGTGCACTTTACATAGGTTTGGTATAGAAAAGGTCTTTATTGACTTTGGAAATACTGA
CCTATGCTTTTACAAACGCTTCAGTTTTAGAATGTCATCCGCGTATAACGCAATAAATACAACTTTCAGC
AACGGATCTCTTGGCTCTCGCATCGATGAAGAACGCAGCGAAATGCGATAAGTAATGTGAATTGCAGAAT
TCAGTGAATCATCGAATCTTTGAACGCATCTTGCGCCCTTTGGTATTCCGAAGGGCATGCCTGTTTGAGT
GTCATGGTATTCTCAATACCCCAAATCTTTGCGGATAAGGGTGTGTTGGACTTGGAGGTTTTTGCAGGTA
ATGATTGTATTACCAGCTCCTCTTAAATGCATTAGCAGAGATAATACTGCTACTCTCCAGTGTGATAATT
GTCTACACTGTTAGTAGTGCGGTATAACAAAATGTCTATGCTTCTAATCGTCTTCGGACAACTTTTGA

Endomelanconiopsis endophytica isolate 10_6

https://www.ncbi.nlm.nih.gov/nuccore/MF773679

ACCGGGGCTGGCCAGCGCCCGCCAGAGGACTACCAAACTCCAGTCAGTAAACGTAGCTGTCTGATCAAAA
GTTTAATAAACTAAAACTTTCAACAACGGATCTCTTGGTTCTGGCATCGATGAAGAACGCAGCGAAATGC
GATAAGTAATGTGAATTGCAGAATTCAGTGAATCATCGAATCTTTGAACGCACATTGCGCCCCTTGGTAT
TCCGAGGGGCATGCCTGTTCGAGCGTCATTTCACCACTCAAGCTCTGCTTGGTATTGGGCGCCGTCCTTC
ACCGGACGCGCCTCAAAGACCTCGGCGGTGGCGTCTTGCCTCAAGCGTAGTAGAAAACACCTCGCTTTGG
AGGACGGGACGTTCGCTCGCCGGACGAACCTTCTGAATTTTCTCAAGGTGACCTCGGATCA

Trichoderma harzianum (11_26)

https://www.ncbi.nlm.nih.gov/nuccore/MF029755.1

ACCTGCGGAGGGATCATTACCGAGTTTACAATCCCAAACCCAATGTGAACGTTACCAAACTGTTGCCTCG
GCGGGATCTCTGCCCCGGGTGCGTCGCAGCCCCGGACCAAGGCGCCCGCCGGAGGACCAACCAAAACTCT
TTTTGTATACCCCCTCGCGGGTTTTTTTATAATCTGAGCCTTCTCGGCGCCTCTAGTAGGCGTTTTGAAA
ATGAATCAAAACTTTCAACAAAGGATCTCTTGGTTTTGGCATCGATGAAGAACGCAGCGAAATGCGATAA
GTAATGTGAATTGCAGAATTCAGTGAATCATAGAATCTATGAACGCACATTGCGCCCGCCAGTATTCCGG
CGGGCATGCCTGTCCAAGCGTCATTTCAACCCTCGAACCCCTCCGGGGGGTCGGCGTTGGGGATCGGCCC
TCCGGGAGCGGGCGGCCGTCTCCGAAATACAGTGGCGGTCTCGCCGCAGCCTGTCATGCGCAGTAGTTTG
CACACTCGCATCGGGAGCGCGGCGCGTCCACAGCCGTTAAACACCCAACTTTGAAATGTGACCTCGGATC

Lasiodiplodia pseudotheobromae (11_11)

https://www.ncbi.nlm.nih.gov/nuccore/MF029754.1

CCTGCGGAAGGATCATTACCGAGTTTTCGGGCTTCGGCTCGACTCTCCCACCCTTTGTGAACGTACCTCT
GTTGCTTTGGCGGCTCCGGCCGCCAAAGGACCTCCAAACTCCAGTCAGTAAACGCAGACGTCTGATAAAC
AAGTTAATAAACTAAAACTTTCAACAACGGATCTCTTGGTTCTGGCATCGATGAAGAACGCAGCGAAATG
CGATAAGTAATGTGAATTGCAGAATTCAGTGAATCATCGAATCTTTGAACGCACATTGCGCCCCTTGGTA
TTCCGGGGGGCATGCCTGTTCGAGCGTCATTACAACCCTCAAGCTCTGCTTGGAATTGGGCACCGTCCTC
ACTGCGGACGCGCCTCAAAGACCTCGGCGGTGGCTGTTCAGCCCTCAAGCGTAGTAGAATACACCTCGCT
TTGGAGTGGTTGGCGTCGCCCGCCGGACGAAC

Sordariomycetes sp. isolate 11_7

https://www.ncbi.nlm.nih.gov/nuccore/MF773680

CCTGCGGAGGGATCATTACTGAGTTATCTAAACTCCAACCCTATGTGAACTTACCGTCGTTGCCTCGGCG
GGCTGCGCTTACCCGGTAGCTACCCTGTAGCTACCCGGTAGCGCGCTACAAAGCCCGCCGGCGGACCACT
AAACTCTGTTATATATACTGTATCTCTGAATGCTTCAAACTTAATAAGTTAAAACTTTCAACAACGGATC
TCTTGGTTCTGGCATCGATGAAGAACGCAGCGAAATGCGATAAGTAATGTGAATTGCAGAATTCAGTGAA
TCATCGAATCTTTGAACGCACATTGCGCCCATTAGTATTCTAGTGGGCATGCCTATTCGAGCGTCATTTC
AACCCTTAAGCCCCTGTTGCTTAGTGTTGGGAATCTGCGTCTTAGGGCGCAGTTCCTCAAAGTGATTGGC
GGAGTTAGGGCATACTCTAAGCGTAGTAATATTCTTCTCGCTTCTGAAGTTGTCCTGGCGGCCGGCCGTA
AAACCCCTATATTTTTAGTGGTGACCTCGAATAG

Nodulisporium sp. (11_8)

https://www.ncbi.nlm.nih.gov/nuccore/MF773681.1

CTGCGGAGGGATCATTACAGAGTTATCCAACTCCCAAACCCATGTGAACATATCTCTTTGTTGCCTCGGC
GCAAGCTACCCGGGACCTCGCGCCCCGGGCGGCCCGCCGGCGGACAAACCAAACTCTGTTATCTTCGTTG
ATTATCTGAGTGTCTTATTTAATAAGTCAAAACTTTCAACAACGGATCTCTTGGTTCTGGCATCGATGAA
GAACGCAGCGAAATGCGATAAGTAATGTGAATTGCAGAATTCAGTGAATCATCGAATCTTTGAACGCACA
TTGCGCCCATTAGTATTCTAGTGGGCATGCCTGTTCGAGCGTCATTTCAACCCCTAAGCACAGCTTATTG
TTGGGCGTCTACGTCTGTAGTGCCTCAAAGACATTGGCGGAGCGGCAGCAGTCCTCTGAGCGTAGTAATT
CTTTATCTCGCTTCTGTTAGGCGCTGCCCCCCCGGCCGTAAAACCCCCA
