# Supplementary material for: Molecular phylogeny and bioprospecting of Endolichenic Fungi (ELF) inhabiting in the lichens collected from a mangrove ecosystem in Sri Lanka
Source: PLoS One. 2018 Aug 29;13(8):e0200711. doi: 10.1371/journal.pone.0200711 (PMC6114277; doi:10.1371/journal.pone.0200711)
Supplement: S2 File — (PDF) [file pone.0200711.s002.pdf]

S2 File. Absorbance values obtain in DPPH assay (at 517nm) for all the ELF isolates used in the study.

DPPH ASSAY

BHT

	1	2	3	MEAN	% inhibition	Dil seres of BHT (	Fiinal con(µg/mL)
A	0.551	0.651	0.665	0.622333	0	0	0
H	0.595	0.638	0.563	0.598667	3.75134	15.62	12.496
G	0.458	0.434	0.598	0.496667	20.15005	31.25	25
F	0.457	0.405	0.349	0.403667	35.10182	62.5	50
E	0.244	0.241	0.233	0.239333	61.52197	125	100
D	0.168	0.256	0.113	0.179	71.22186	250	200
C	0.134	0.137	0.162	0.144333	76.79528	500	400
B	0.114	0.113	0.185	0.137333	77.92069	1000	800

N/L5/E15			Mean	%
0.6020	0.6073	0.6151	0.6081	
0.5899	0.5817	0.5966	0.5894	
0.5834	0.5740	0.5755	0.5776	
0.5654	0.5584	0.5660	0.5633	
0.5168	0.5448	0.5334	0.5317	
0.4926	0.4970	0.4997	0.4964	
0.3405	0.3387	0.3613	0.3468	
0.1435	0.1171	0.1173	0.1260	

N/L7/E3			Mean
0.5182	0.5172	0.5180	0.5178
0.5160	0.5152	0.5146	0.5153
0.4451	0.4551	0.4445	0.4482
0.3100	0.3190	0.3078	0.3123
0.2506	0.2743	0.2840	0.2696
0.2101	0.2139	0.2108	0.2116
0.1806	0.1688	0.1732	0.1742
0.1443	0.1487	0.1471	0.1467

N/L4/E11			Mean
0.6049	0.6052	0.6003	0.6035
0.4754	0.4710	0.4863	0.4776
0.3630	0.3917	0.3853	0.3800
0.2266	0.2352	0.1994	0.2204
0.1246	0.1311	0.1234	0.1264
0.1058	0.1032	0.1006	0.1032
0.0972	0.0988	0.0968	0.0976
0.0899	0.0888	0.0989	0.0925

AT-11/L6/E5			Mean
0.6029	0.6162	0.6102	0.6098
0.5765	0.5649	0.5616	0.5677
0.5219	0.5445	0.5461	0.5375
0.4957	0.5071	0.4963	0.4997
0.3096	0.3013	0.3335	0.3148
0.2122	0.2969	0.2927	0.2673
0.1205	0.1515	0.1685	0.1468
0.1118	0.1153	0.1204	0.1158

AT/L3/E1			Mean
0.7635	0.7678	0.7276	0.7530
0.5521	0.5479	0.4971	0.5324
0.4052	0.4275	0.3318	0.3882
0.1946	0.2443	0.1931	0.2107
0.1655	0.1621	0.1620	0.1632
0.0955	0.0929	0.0836	0.0907
0.0735	0.0838	0.0711	0.0761
0.0549	0.0649	0.0462	0.0553

AT/L11/E1			Mean
0.7323	0.7290	0.7217	0.7277
0.7063	0.7288	0.7418	0.7256
0.7061	0.7095	0.7092	0.7083
0.6901	0.6933	0.6992	0.6942
0.6207	0.6236	0.6286	0.6243
0.5900	0.5813	0.6006	0.5906
0.4641	0.4150	0.4746	0.4512
0.2476	0.2399	0.2460	0.2445

AT/L12/E2			Mean
0.8176	0.8339	0.7663	0.8059
0.7924	0.7724	0.7292	0.7647
0.7708	0.7922	0.6674	0.7435
0.7397	0.7387	0.7047	0.7277
0.6782	0.6780	0.6441	0.6668
0.5840	0.5680	0.5527	0.5682
0.3821	0.4203	0.3968	0.3997
0.3348	0.3759	0.2656	0.3254

AT/L6/5			Mean
0.7876	0.8080	0.8052	0.8003
0.7811	0.8169	0.8146	0.8042
0.7952	0.7826	0.7886	0.7888
0.7696	0.7554	0.7435	0.7562
0.7438	0.7440	0.7466	0.7448
0.6723	0.6766	0.6733	0.6741
0.5761	0.5775	0.5833	0.5790
0.4041	0.4153	0.4080	0.4091

AT/L5/E1			Mean
0.8561	0.8251	0.8438	0.8417
0.7986	0.7827	0.8470	0.8094
0.7956	0.7483	0.8031	0.7823
0.7245	0.8446	0.6904	0.7532
0.7035	0.7170	0.7343	0.7183
0.6668	0.6796	0.7290	0.6918
0.6109	0.5477	0.6671	0.6086
0.5854	0.5753	0.6256	0.5954

AT/L9/E1			Mean
0.7404	0.7446	0.7724	0.7525
0.4807	0.4816	0.4239	0.4621
0.4330	0.3389	0.4323	0.4014
0.2925	0.2944	0.3060	0.2976
0.2203	0.2633	0.2642	0.2493
0.1929	0.1883	0.1937	0.1916
0.1698	0.1517	0.1709	0.1641
0.1472	0.1458	0.1561	0.1497

NT/L2/E1			Mean
0.8019	0.7075	0.8127	0.7740
0.7988	0.7104	0.7935	0.7676
0.7621	0.6965	0.7538	0.7375
0.6894	0.6421	0.6967	0.6761
0.5534	0.4949	0.5683	0.5389
0.3230	0.3114	0.3366	0.3237
0.1763	0.1716	0.1900	0.1793
0.1532	0.1469	0.1560	0.1520

	AT/L13/E2		Mean
0.7875	0.8764	0.7689	0.8109
0.7889	0.8135	0.7928	0.7984
0.7932	0.8288	0.7645	0.7955
0.7429	0.8261	0.7431	0.7707
0.7168	0.7729	0.7138	0.7345
0.6097	0.6840	0.6164	0.6367
0.4886	0.5339	0.4821	0.5015
0.2729	0.3235	0.2892	0.2952

	N/L4/E4		Mean
0.7415	0.8100	0.7791	0.7769
0.7952	0.7921	0.7250	0.7708
0.7526	0.7890	0.7360	0.7592
0.6535	0.6662	0.6487	0.6561
0.4583	0.5121	0.4962	0.4889
0.3373	0.3383	0.3136	0.3297
0.2324	0.2290	0.1775	0.2130
0.1444	0.1571	0.1474	0.1496

	N/L8/E1		Mean
0.8924	0.8760	0.9284	0.8989
0.9015	0.8641	0.8958	0.8871
0.8708	0.8272	0.8329	0.8436
0.8459	0.8197	0.8608	0.8421
0.8671	0.6439	0.8426	0.7845
0.7576	0.8554	0.7124	0.7751
0.7218	0.6895	0.7494	0.7202
0.5327	0.5063	0.5488	0.5293

AT/L11/E2			Mean
0.8784	0.8676	0.8460	0.8640
0.8583	0.8863	0.8149	0.8532
0.8718	0.8804	0.8022	0.8515
0.8394	0.8402	0.7975	0.8257
0.7958	0.8215	0.7417	0.7863
0.7556	0.7297	0.7227	0.7360
0.6048	0.6380	0.5617	0.6015
0.4556	0.4587	0.4277	0.4473

N/L9/E1			Mean
0.8897	0.8602	0.9155	0.8885
0.8689	0.8553	0.8892	0.8711
0.8602	0.8423	0.8959	0.8661
0.8536	0.8345	0.8561	0.8481
0.8105	0.7771	0.8195	0.8024
0.7531	0.7368	0.7618	0.7506
0.6538	0.6448	0.6682	0.6556
0.5504	0.5769	0.5261	0.5511

AT-11/L6/E1			Mean
0.7538	0.7451	0.7264	0.7418
0.7212	0.7373	0.7290	0.7292
0.6766	0.6801	0.6830	0.6799
0.7382	0.6608	0.6371	0.6787
0.6575	0.6438	0.6356	0.6456
0.5774	0.5884	0.5580	0.5746
0.4160	0.3978	0.4181	0.4106
0.1244	0.1182	0.1162	0.1196

N/L10/E4*			Mean
0.6577	0.6871	0.6705	0.6718
0.6557	0.6400	0.6186	0.6381
0.6005	0.6362	0.6314	0.6227
0.5374	0.5396	0.5637	0.5469
0.3575	0.3486	0.3609	0.3557
0.1370	0.1380	0.1453	0.1401
0.1194	0.1117	0.1230	0.1180
0.1136	0.1014	0.1009	0.1053

AT-11/L6/E3			Mean
0.7636	0.7362	0.7385	0.7461
0.7489	0.7113	0.7450	0.7351
0.7192	0.7378	0.7281	0.7284
0.6965	0.7497	0.6968	0.7143
0.7025	0.7101	0.6931	0.7019
0.6275	0.6230	0.6203	0.6236
0.5613	0.5612	0.5614	0.5613
0.5012	0.4956	0.4865	0.4944

N/L4/E17			Mean
0.9758	0.8613	1.0091	0.9487
0.9213	0.8817	0.9261	0.9097
0.8907	0.8954	0.8850	0.8904
0.8168	0.8087	0.8467	0.8241
0.7122	0.7726	0.8633	0.7827
0.6604	0.6102	0.6165	0.6290
0.3156	0.2367	0.4219	0.3247
0.1998	0.1762	0.1697	0.1819

	N/L1/E3		Mean
1.0471	0.9268	0.9725	0.9821
1.0054	0.9127	1.0132	0.9771
0.9818	0.9213	0.1571	0.6867
0.9756	0.8763	0.9365	0.9295
0.9598	0.7405	0.7131	0.8045
0.9655	0.8177	0.5893	0.7908
0.9647	0.9035	0.6847	0.8510
0.9431	0.1102	0.5515	0.5349

	AT/L11/E3		Mean
0.8922	0.8986	0.9203	0.904
0.9800	1.0160	0.8381	0.945
0.9134	0.9643	0.8855	0.921
0.9183	0.9076	0.9196	0.915
0.4140	0.9325	0.7754	0.707
0.4630	0.9089	0.8311	0.734
0.5213	0.8127	0.7457	0.693
0.9253	0.8044	0.6998	0.810

	7/30		Mean
0.7703	0.7642	0.7749	0.7698
0.7487	0.7675	0.7482	0.7548
0.7255	0.7124	0.7236	0.7205
0.7143	0.7126	0.7045	0.7105
0.6827	0.6918	0.6968	0.6904
0.6739	0.6778	0.6765	0.6761
0.6168	0.6215	0.6177	0.6187
0.5044	0.5535	0.6106	0.5562

	10/6			Mean
0.7996	0.7799		0.8193	0.7996
0.7890	0.7807		0.7888	0.7862
0.7873	0.7866		0.7871	0.7870
0.7747	0.7614		0.7717	0.7693
0.6987	0.6999		0.6988	0.6991
0.6969	0.6900		0.6852	0.6907
0.6439	0.6510		0.6520	0.6490
0.6990	0.6113		0.6011	0.6371

	11/8		Mean
0.7526	0.8043	0.7890	0.7820
0.7962	0.8017	0.7965	0.7981
0.7889	0.7969	0.7821	0.7893
0.7999	0.7503	0.7591	0.7698
0.6866	0.6890	0.6999	0.6918
0.6744	0.6760	0.6800	0.6768
0.6450	0.6461	0.6491	0.6467
0.6565	0.6585	0.6633	0.6594

	7/18		Mean
0.8189	0.8188	0.8310	0.8229
0.7910	0.7875	0.7947	0.7911
0.7882	0.7888	0.7817	0.7862
0.7646	0.7609	0.7671	0.7642
0.7018	0.7162	0.7007	0.7062
0.6693	0.6965	0.6639	0.6766
0.6235	0.6632	0.6703	0.6523
0.6122	0.6200	0.6169	0.6164

	N/L7/E1		Mean
0.8209	0.8211	0.8382	0.8267
0.8196	0.8200	0.8211	0.8202
0.7867	0.7856	0.7866	0.7863
0.7300	0.7211	0.7100	0.7204
0.6179	0.6132	0.6218	0.6176
0.5868	0.5867	0.5768	0.5834
0.4392	0.4872	0.4708	0.4657
0.3184	0.3617	0.3761	0.3521

	8/14		Mean
0.8546	0.8641	0.8550	0.8579
0.8493	0.8427	0.8518	0.8479
0.8320	0.8380	0.8504	0.8401
0.7662	0.7466	0.7399	0.7509
0.7450	0.7415	0.7418	0.7428
0.7397	0.7400	0.7419	0.7405
0.7293	0.7266	0.7295	0.7285
0.7182	0.7198	0.7984	0.7455

	AT/L1/E6			
(µg/mL)	Trial1	Trial 2	Trial2	mean
0	0	0	0	0
12.496	3.110889	2.6108886	3.114889	2.945555251
25	4.148765	3.758765	4.258765	4.055431643
50	12.13728	11.987276	11.98728	12.03727557
100	16.49763	17.692628	17.69263	17.294295
200	25.69523	26.895232	24.99523	25.86189874
400	57.7707	55.978704	56.9587	56.90270377
800	72.82639	70.829394	71.95939	71.87172735

AT/L1/E7			
Trial1	Trial 2	Trial2	mean
0	0	0	0
2.310689	2.610889	2.454889	2.458822
3.348765	2.958765	3.216259	3.174596
9.127276	8.987276	7.987276	8.700609
13.49763	12.69263	13.69263	13.2943
22.79523	22.89423	23.49523	23.06157
55.5707	54.9887	53.8587	54.80604
71.82639	70.82939	69.95939	70.87173

6/17			
Trial1	Trial 2	Trial2	mean
0	0	0	0
3.610889	3.610889	3.555889	3.592555
4.148765	4.125876	4.258765	4.177802
10.13728	10.28728	10.18728	10.20394
15.49763	15.49263	15.39263	15.46096
25.69523	25.89523	25.99523	25.8619
53.1707	53.1787	53.1587	53.16937
70.12639	70.12939	71.15939	70.47173

AT/L1/E1*			
Trial1	Trial 2	Trial2	mean
0	0	0	0
0.83118	1.00118	1.02118	0.951180017
2.720069	3.1520069	2.980069	2.950714901
8.652885	9.0028854	8.652885	8.76955211
15.37898	15.378984	16.17898	15.6456503
28.1826	29.782601	27.1826	28.38260121
53.83463	52.834625	51.83463	52.83462532
69.35745	70.35745	72.95745	70.89078381

	AT/L6/E1*		
Trial1	Trial 2	Trial2	mean
0	0	0	0
1.83118	1.00118	1.02118	1.284513
2.720069	3.152007	2.980069	2.950715
10.25289	9.002885	10.15289	9.802885
16.37898	14.37898	16.17898	15.64565
30.1826	31.7826	30.1826	30.71593
56.13463	54.23463	55.93463	55.43463
73.35745	73.35745	75.95745	74.22412

	N/L4/E23		
Trial1	Trial 2	Trial2	mean
0	0	0	0
1.63118	1.40118	1.52118	1.284513
2.720069	2.152007	2.980069	2.950715
9.152885	9.502885	8.752885	9.802885
14.37898	14.37898	13.27898	15.64565
26.1826	25.7826	24.1826	30.71593
53.13463	54.23463	51.93463	55.43463
75.15745	74.25745	73.12557	74.22412

N/L7/E3			Mean
0.5182	0.5172	0.518	0.5178
0.516	0.5152	0.5146	0.515266667
0.4451	0.4551	0.4445	0.448233333
0.31002	0.319	0.3078	0.312273333
0.2506	0.2743	0.284	0.269633333
0.2101	0.2139	0.2108	0.2116
0.1806	0.1688	0.1732	0.1742
0.1443	0.1487	0.1471	0.1467

AT/L2/E2			Mean
0	0	0	0
1.53118	1.20118	1.32118	1.35118
2.720069	3.152007	2.980069	2.950715
10.25289	9.002885	10.15289	9.802885
17.37898	17.37898	16.87898	17.21232
47.1826	46.7826	48.1826	47.3826
56.13463	54.23463	55.93463	55.43463
71.35745	72.31745	72.15745	71.94412

AT/L4/E3			Mean
0	0	0	0
1.816433	2.316433	2.116433	2.0831
3.147032	3.547032	3.347032	3.347032
10.20344	9.703437	10.20344	10.03677
37.26434	36.76434	37.16434	37.06434
53.49084	53.12084	52.99084	53.20084
63.19914	64.19914	64.39914	63.93247
73.11865	72.61865	73.21865	72.98531

AT/L5/E4			mean
0	0	0	0
1.516433	1.316433	1.216433	1.349766307
2.547032	2.4470323	3.117032	2.70369897
8.203437	9.1034372	8.253437	8.520103818
30.16434	30.764342	30.21434	30.38100881
43.49084	44.520838	46.19084	44.73417173
60.79914	61.19914	61.39914	61.13247301
70.11865	72.018646	71.21865	71.11864582

AT/L6/E10

(μg/mL)	Trial1	Trial 2	Trial2	mean
0	0	0	0	0
12.496	1.2408886	1.310759	1.113488584	1.221712
25	2.258765	2.314765	3.112664977	2.562065
50	12.137276	12.51424	12.18627757	12.27926
100	24.497628	24.21433	23.75281833	24.15493
200	36.195232	36.89523	36.99523207	36.69523
400	57.770704	55.9787	56.95870377	56.9027
800	72.826394	70.82939	71.95939401	71.87173

AT/L6/E12

(μg/mL)	Trial1	Trial 2	Trial2	mean
0	0	0	0	0
12.496	1.7030646	1.703065	1.703064618	1.703065
25	2.5465673	2.344567	2.357567269	2.416234
50	3.5063359	3.116436	3.25433594	3.292369
100	12.963962	12.96396	12.96396154	12.96396
200	22.539768	22.53977	22.53976813	22.53977
400	46.34366	45.94616	45.71565957	46.00183
800	68.177056	67.97605	68.98405581	68.37905

(μg/mL)	AT/L7/E1			mean
0	0	0	0	0
12.496	1.816433	2.316433	2.116432973	2.0831
25	3.1470323	3.547032	3.347032303	3.347032
50	10.203437	9.703437	10.20343715	10.03677
100	22.264342	23.76434	23.66434214	23.23101
200	44.490838	43.12084	43.1908384	43.60084
400	63.19914	64.19914	64.39913968	63.93247
800	71.118646	71.61865	72.21864582	71.65198

AT/L8/E1

(μg/mL)	Trial1	Trial 2	Trial2	mean
0	0	0	0	0
12.496	1.1108886	0.910889	1.114888584	1.045555
25	2.148765	2.758765	2.258764977	2.388765
50	6.1372756	5.987276	5.78727557	5.970609
100	14.497628	13.69263	13.77262833	13.98763
200	30.695232	28.89523	29.99523207	29.8619
400	55.770704	55.9787	55.95870377	55.9027
800	68.826394	70.82939	67.95939401	69.20506

AT/L8/E5

(μg/mL)	Trial1	Trial 2	Trial2	mean
0	0	0	0	0
12.496	39.83118	39.86118	38.79518002	39.49585
25	47.590069	47.67407	47.55706891	47.60707
50	62.152885	62.14289	63.11888544	62.47155
100	67.378984	67.17898	66.57898363	67.04565
200	75.182601	75.1826	75.18260121	75.1826
400	79.134625	79.23463	79.33462532	79.23463
800	80.35745	80.35745	80.35745047	80.35745

AT/L8/E12

(μg/mL)	Trial1	Trial 2	Trial2	mean
0	0	0	0	0
12.496	1.7030646	1.703065	1.703064618	1.703065
25	2.1465673	2.314567	2.304567269	2.255234
50	3.1063359	3.106436	3.10333594	3.105369
100	10.963962	10.86396	11.15396154	10.99396
200	22.539768	22.53977	22.53976813	22.53977
400	47.34366	47.94616	47.71565957	47.66849
800	65.177056	64.97605	64.98405581	65.04572

(µg/mL)	Trial1	Trial 2	Trial2	mean
0	0	0	0	0
12.496	1.416433	1.616433	1.356432973	1.4631
25	2.7470323	2.654032	2.857032303	2.752699
50	10.253437	10.98344	10.16343715	10.46677
100	25.664342	25.76434	26.11434214	25.84768
200	44.590838	44.72084	46.1908384	45.16751
400	62.19914	61.19914	61.39913968	61.59914
800	73.118646	72.01865	72.21864582	72.45198

	AT/L11/E3			
(µg/mL)	Trial1	Trial 2	Trial2	mean
0	0	0	0	0
12.496	0.7030646	0.973065	1.001064618	0.892398
25	1.5145673	1.485567	1.409456727	1.469864
50	2.6463336	2.966336	2.868763359	2.827144
100	10.163962	10.98596	10.15426154	10.43473
200	25.539768	26.13977	25.64576813	25.7751
400	40.14366	41.15366	41.98665957	41.09466
800	65.877056	64.96806	65.55140558	65.46551

	AT/L11/E1*			
(µg/mL)	Trial1	Trial 2	Trial2	mean
0	0.0045806	0.004581	0.004580642	0.004581
12.496	0.2839998	0.284	0.283999817	0.284
25	2.6705144	2.670514	2.670514406	2.670514
50	4.6035454	4.603545	4.603545417	4.603545
100	14.209152	14.20915	14.20915212	14.20915
200	18.835601	18.8356	18.83560075	18.8356
400	37.991846	37.99185	37.99184646	37.99185
800	66.400989	66.40099	66.40098942	66.40099

(µg/mL)	AT/L12/E4*			mean
0	0	0	0	0
12.496	1.816433	2.316433	2.116432973	2.0831
25	3.1470323	3.547032	3.347032303	3.347032
50	10.203437	9.703437	10.20343715	10.03677
100	20.264342	19.76434	20.16434214	20.06434
200	38.490838	37.12084	37.1908384	37.60084
400	58.19914	57.99914	59.39913968	58.53247
800	70.118646	70.61865	71.21864582	70.65198

	NT/L1/E1			
(µg/mL)	Trial1	Trial 2	Trial2	mean
0	0	0	0	0
12.496	35.195792	34.89579	35.18579181	35.09246
25	41.157807	40.87981	41.13780731	41.05847
50	57.147398	56.9874	56.98647398	57.04042
100	63.175862	63.16486	62.87486157	63.07186
200	71.533776	70.83378	71.6438763	71.33714
400	75.978261	75.87829	76.18826135	76.01494
800	78.106312	78.27631	77.70631229	78.02965

	NT/L1/E3			
(µg/mL)	Trial1	Trial 2	Trial2	mean
0	0	0	0	0
12.496	1.416433	1.616433	1.356432973	1.4631
25	2.7470323	2.654032	2.857032303	2.752699
50	9.2534372	9.983437	8.963437151	9.400104
100	27.664342	27.76434	26.11434214	27.18101
200	46.590838	46.72084	47.1908384	46.83417
400	65.19914	66.19914	65.39913968	65.59914
800	71.118646	71.01865	72.21864582	71.45198
