# Supplementary material for: Molecular phylogeny and bioprospecting of Endolichenic Fungi (ELF) inhabiting in the lichens collected from a mangrove ecosystem in Sri Lanka
Source: PLoS One. 2018 Aug 29;13(8):e0200711. doi: 10.1371/journal.pone.0200711 (PMC6114277; doi:10.1371/journal.pone.0200711)
Supplement: S4 File — (PDF) [file pone.0200711.s004.pdf]

S4 File. Absorbance values obtain in amylase inhibitory assay (at 540nm) for all the ELF isolates used in the study

Sample ID		Absorbance trial 1	% inhibition trial 1	Absorbance trial 2	% inhibition trial 2	Absorbance trial 3	% inhibition trial 3
	control 1	0.348		0.348		0.355	
		0.130	62.66	0.130	62.650	0.135	62.66
AT/L1/E6	Schizophyllum commune	0.335	3.756	0.339	2.568	0.308	5.034
AT/L1/E1*	Diaporthe arengae	0.305	12.456	0.303	12.989	0.218	14.095
AT/L1/E7	Schizophyllum commune	0.336	3.569	0.334	4.021	0.298	6.096
AT/L2/E2	Aspergillus hiratsukae	0.339	2.672	0.338	2.995	0.338	2.016
AT/L3/E1	Sordaria sp.	0.322	7.568	0.324	7.023	0.287	7.123
AT/L4/E3	Lasiodiplodia theobromae	0.306	11.985	0.304	12.652	0.215	14.321
AT/L5/E4	Lasiodiplodia theobromae	0.302	13.258	0.298	14.235	0.185	17.333
AT/L6/E5	Hypoxylon anthochroum	0.313	10.001	0.310	11.023	0.269	8.976
AT/L6/E10	Aspergillus hiratsukae	0.337	3.254	0.338	2.956	0.323	3.585
AT/L6/E12	Nigrospora sphaerica	0.295	15.259	0.292	16.012	0.185	17.386
AT/L6/E1*	Diaporthe arengae	0.298	14.325	0.301	13.624	0.218	14.087
AT-II/L6/E3	Xylaria feejeensis	0.280	19.523	0.283	18.625	0.149	20.994

AT-II/L6/E1	Chaetomium fuscum	0.341	1.998	0.341	2.014	0.312	4.661
AT-II/L6/E5	Preussia sp.	0.300	13.689	0.297	14.562	0.204	15.426
AT/L7/E1	Neurospora crassa	0.246	29.358	0.231	33.523	-0.059	41.714
AT/L8/E1	Neurospora sp	0.242	30.562	0.266	23.568	0.015	34.373
AT/L8/E5	Daldinia eschscholtzii	0.248	28.698	0.260	25.369	0.033	32.522
AT/L8/E12	Cerrena unicolor	0.343	1.539	0.344	1.035	0.336	2.298
AT/L9/E1	Daldinia eschscholtzii	0.208	40.123	0.218	37.259	-0.059	41.775
AT/L11/E3 *	Lasiodiplodia theobromae	0.294	15.568	0.296	14.985	0.189	16.955
AT/L11/E1	Endomelanconiops is endophytica	0.249	28.368	0.252	27.456	-0.006	36.459
AT/L11/E3	Neosartorya hiratsukae	0.329	5.59	0.327	6.024	0.268	9.074
AT/L12/E2	Neosartorya hiratsukae	0.331	4.998	0.329	5.328	0.293	6.531
AT/L12/E4 *	Neurospora crassa	0.208	40.325	0.215	38.125	0.012	34.692
AT/L13/E2	Xylaria psidii	0.313	10.012	0.316	9.258	0.274	8.42
NT/L1/E1	Daldinia eschscholtzi	0.296	15.014	0.298	14.328	0.209	14.941
NT/L1/E3	Lasiodiplodia theobromae	0.316	9.214	0.312	10.254	0.228	13.097
NT/L2/E1	Daldinia eschscholtzii	0.244	29.895	0.243	30.254	0.024	33.463
NT/L3/E1	Daldinia eschscholtzii	0.248	28.698	0.254	26.998	0.083	27.59
N/L1/E3	Xylariaceae sp	0.262	24.658	0.253	27.321	0.138	22.019

N/L2/E4	Daldinia eschscholtzii	0.259	25.698	0.265	23.856	0.061	29.754
N/L2/E7	Xylaria castorea	0.324	6.789	0.323	7.234	0.269	8.93
N/L4/E11	Diaporthe musigena	0.311	10.512	0.313	9.996	0.257	10.122
N/L4/E23	Diaporthe arengae	0.305	12.256	0.306	11.995	0.235	12.358
N/L5/E2	Daldinia sp	0.267	23.156	0.275	20.896	0.121	23.787
N/L6/E1	Preussia tenerifae	0.269	22.561	0.263	24.354	0.140	21.815
N/L7/E3	Nigrospora sp.	0.331	4.998	0.331	5.002	0.299	5.963
N/L7/E6	Rigidoporus vinctus	0.324	6.982	0.329	5.468	0.293	6.531
N/L8/E2	Lasiodiplodia theobromae	0.321	7.658	0.320	8.001	0.256	10.21
N/L8/E1	Lasiodiplodia pseudotheobromae	0.299	14.214	0.299	13.998	0.219	13.932
N/L9/E1	Lasiodiplodia theobromae	0.324	6.881	0.326	6.315	0.284	7.444
N/L9/E4	Daldinia eschscholtzii	0.263	24.398	0.264	23.998	0.069	28.998
N/L10/E1*	Lasiodiplodia theobromae	0.322	7.598	0.320	8.001	0.269	8.92
N/L10/E4*	Byssochlamys spectabilis	0.338	3.001	0.339	2.698	0.338	2.014
N/L10/E1	Chaetomium sp	0.338	3.012	0.338	3.001	0.322	3.617
KO2_23	Daldinia eschscholtzii	0.244	29.987	0.243	30.125	0.043	31.574
KO2_7	Hypoxylon anthochroum	0.259	25.68	0.266	23.456	0.173	18.583
KO4_22	Lasiodiplodia crassispora	0.315	9.358	0.320	7.985	0.273	8.526

KO4_34	Daldinia eschscholtzii	0.248	28.645	0.254	26.989	0.076	28.246
KO5_30	Daldinia eschscholtzii	0.270	22.358	0.268	23.014	0.100	25.89
KO5_8	Nodulisporium sp	0.277	20.321	0.271	22.235	0.209	14.915
KO5_12	Hypoxylon anthochroum	0.301	13.568	0.304	12.654	0.207	15.157
KO6_12	Xylariaceae sp	0.296	15.023	0.296	14.998	0.190	16.875
KO6_17	Schizophyllum commune	0.329	5.325	0.326	6.231	0.305	5.307
KO7_26	Endomelanconiopsis endophytica	0.248	28.863	0.244	30.025	0.027	33.125
KO7_2	Daldinia eschscholtzii	0.252	27.651	0.255	26.721	0.060	29.904
KO7_18	Aspergillus aculeatus	0.296	14.985	0.296	15.023	0.195	16.351
KO7_30	Talaromyces pinophilus	0.334	3.995	0.334	4.012	0.313	4.596
KO8_8	Endomelanconiopsis sp	0.237	32.023	0.243	30.125	0.002	35.634
KO8_19	Phomopsis sp.	0.298	14.256	0.296	15.014	0.194	16.423
KO9_10	Cerrena sp	0.341	1.982	0.341	1.998	0.329	2.95
KO10_6	Endomelanconiopsis sp.	0.317	9.025	0.317	8.975	0.255	10.35
KO10_9	Cerrena sp.	0.334	3.948	0.338	2.989	0.330	2.867
KO11_26	Trichoderma harzianum	0.344	1.065	0.345	1.004	0.332	2.62
KO11_11	Lasiodiplodia pseudotheobromae	0.309	11.254	0.310	10.998	0.232	12.644
KO11_7	Sordariomycetes sp.	0.344	1.22	0.345	0.991	0.341	1.749

KO11_8	Nigrospora sphaerica	0.328	5.789	0.330	5.036	0.298	6.032
