# Supplementary material for: Molecular phylogeny and bioprospecting of Endolichenic Fungi (ELF) inhabiting in the lichens collected from a mangrove ecosystem in Sri Lanka
Source: PLoS One. 2018 Aug 29;13(8):e0200711. doi: 10.1371/journal.pone.0200711 (PMC6114277; doi:10.1371/journal.pone.0200711)
Supplement: S5 File — (DOCX) [file pone.0200711.s005.docx]

Schizophyllum commune (AT_L1_E6)

<https://www.ncbi.nlm.nih.gov/nuccore/MF773651.1>

Schizophyllum commune (AT_L1_E7)

<https://www.ncbi.nlm.nih.gov/nuccore/MF773657.1>

Diaporthe arengae (AT_L1_E1ST)

<https://www.ncbi.nlm.nih.gov/nuccore/MF773656.1>

Aspergillus hiratsukae (AT_L2_E2)

<https://www.ncbi.nlm.nih.gov/nuccore/MG593848.1>

Lasiodiplodia theobromae (AT_L4_E3)

<https://www.ncbi.nlm.nih.gov/nuccore/KY992568.1>

Lasiodiplodia theobromae (AT_L5_E4)

<https://www.ncbi.nlm.nih.gov/nuccore/KY992574.1>

Sordaria sp. (AT_L3_E1)

<https://www.ncbi.nlm.nih.gov/nuccore/KY992583.1>

Hypoxylon anthochroum (AT_L6_E5)

<https://www.ncbi.nlm.nih.gov/nuccore/KY992584.1>

Aspergillus hiratsukae (AT_L6_E10)

<https://www.ncbi.nlm.nih.gov/nuccore/KY977718.1>

Nigrospora sphaerica (AT_L6_E12)

<https://www.ncbi.nlm.nih.gov/nuccore/KY977719.1>

Xylaria feejeensis (ATII_L6_E1)

<https://www.ncbi.nlm.nih.gov/nuccore/MG593846.1>

Chaetomium fuscum (ATII_L6_E1)

<https://www.ncbi.nlm.nih.gov/nuccore/1187200607>

Preussia sp. (ATII_L6_E5)

<https://www.ncbi.nlm.nih.gov/nuccore/KY992581>

Neurospora crassa (AT_L7_E1)

<https://www.ncbi.nlm.nih.gov/nuccore/KY992579.1>

Nigrospora sp. (AT_L8_E1)

<https://www.ncbi.nlm.nih.gov/nuccore/KY992575.1>

Daldinia eschscholtzii (AT_L8_E5)

<https://www.ncbi.nlm.nih.gov/nuccore/KY977720.1>

Cerrena unicolor (AT_L8_E12)

<https://www.ncbi.nlm.nih.gov/nuccore/KY977721.1>

Daldinia eschscholtzii (AT_L9_E1)

<https://www.ncbi.nlm.nih.gov/nuccore/MF773660.1>

Aspergillus hiratsukae (AT_L11_E3)

<https://www.ncbi.nlm.nih.gov/nuccore/KY977724.1>

Endomelanconiopsis endophytica (AT_L11_E1)

<https://www.ncbi.nlm.nih.gov/nuccore/KY977723.1>

Aspergillus hiratsukae (AT_L12_E2)

<https://www.ncbi.nlm.nih.gov/nuccore/KY977725.1>

Neurospora crassa (AT_L12_E4ST)

<https://www.ncbi.nlm.nih.gov/nuccore/KY992573>

Xylaria psidii (AT_L13_E2)

<https://www.ncbi.nlm.nih.gov/nuccore/MF773655.1>

Daldinia eschscholtzii (NT_L1_E1)

<https://www.ncbi.nlm.nih.gov/nuccore/KY992578.1>

Lasiodiplodia theobromae (NT_L1_E3)

<https://www.ncbi.nlm.nih.gov/nuccore/KY977731.1>

Daldinia eschscholtzii (NT_L2_E1)

<https://www.ncbi.nlm.nih.gov/nuccore/KY992576.1>

Daldinia eschscholtzii (NT_L3_E1)

<https://www.ncbi.nlm.nih.gov/nuccore/MF773669.1>

Xylariaceae sp. (N_L1_E3)

<https://www.ncbi.nlm.nih.gov/nuccore/MF773661.1>

Daldinia eschscholtzii (N_L2_E4)

<https://www.ncbi.nlm.nih.gov/nuccore/MF773663.1>

Xylaria castorea (N_L2_E7)

<https://www.ncbi.nlm.nih.gov/nuccore/MF773662.1>

Diaporthe musigena (N_L4_E11)

<https://www.ncbi.nlm.nih.gov/nuccore/KY977726.1>

Diaporthe arengae (N_L4_E23)

<https://www.ncbi.nlm.nih.gov/nuccore/KY977727.1>

Daldinia sp. (N_L5_E2)

<https://www.ncbi.nlm.nih.gov/nuccore/MF773665.1>

Preussia tenerifae (N_L6_E1)

<https://www.ncbi.nlm.nih.gov/nuccore/KY992582.1>

Nigrospora sp. (N_L7_E3)

<https://www.ncbi.nlm.nih.gov/nuccore/KY992566.1>

Rigidoporus vinctus (N_L7_E6)

<https://www.ncbi.nlm.nih.gov/nuccore/KY992567.1>

Lasiodiplodia theobromae (N_L8_E2)

<https://www.ncbi.nlm.nih.gov/nuccore/KY992571.1>

Lasiodiplodia pseudotheobromae (N_L8_E1)

<https://www.ncbi.nlm.nih.gov/nuccore/KY977728.1>

Lasiodiplodia theobromae (N_L9_E1)

<https://www.ncbi.nlm.nih.gov/nuccore/KY992570.1>

Daldinia eschscholtzii (N_L9_E4)

<https://www.ncbi.nlm.nih.gov/nuccore/KY977729.1>

Byssochlamys spectabilis (N_L10_E4ST)

<https://www.ncbi.nlm.nih.gov/nuccore/KY977730>

Chaetomium sp. (N_L10_E1)

<https://www.ncbi.nlm.nih.gov/nuccore/MF773667.1>

Daldinia eschscholtzii (2_23)

<https://www.ncbi.nlm.nih.gov/nuccore/MF773682.1>

Hypoxylon anthochroum (2_7)

<https://www.ncbi.nlm.nih.gov/nuccore/KY985428>

Daldinia eschscholtzii (4_34)

<https://www.ncbi.nlm.nih.gov/nuccore/MF029744.1>

Lasiodiplodia crassispora (4_22)

<https://www.ncbi.nlm.nih.gov/nuccore/MF029743.1>

Daldinia eschscholtzii (5_30)

<https://www.ncbi.nlm.nih.gov/nuccore/MF029745.1>

Hypoxylon anthochroum (5_12)

<https://www.ncbi.nlm.nih.gov/nuccore/MF773672.1>

Xylariaceae sp. (6_12)

<https://www.ncbi.nlm.nih.gov/nuccore/MF029748.1>

Schizophyllum commune (6_17)

<https://www.ncbi.nlm.nih.gov/nuccore/MF773673.1>

Daldinia eschscholtzii isolate 7_2

<https://www.ncbi.nlm.nih.gov/nuccore/MF773674.1>

Endomelanconiopsis sp. (8_8)

<https://www.ncbi.nlm.nih.gov/nuccore/MF029751.1>

Endomelanconiopsis endophytica (7_26)

<https://www.ncbi.nlm.nih.gov/nuccore/MF029750.1>

Aspergillus aculeatus (7_18)

<https://www.ncbi.nlm.nih.gov/nuccore/MF773675.1>

Talaromyces pinophilus (7_19)

<https://www.ncbi.nlm.nih.gov/nuccore/MF773676>

Phomopsis sp. (8_19)

<https://www.ncbi.nlm.nih.gov/nuccore/MF773677.1>

Cerrena sp. (9_10)

<https://www.ncbi.nlm.nih.gov/nuccore/MF773678.1>

Endomelanconiopsis endophytica isolate 10_6

<https://www.ncbi.nlm.nih.gov/nuccore/MF773679>

Trichoderma harzianum (11_26)

<https://www.ncbi.nlm.nih.gov/nuccore/MF029755.1>

Lasiodiplodia pseudotheobromae (11_11)

<https://www.ncbi.nlm.nih.gov/nuccore/MF029754.1>

Sordariomycetes sp. isolate 11_7

<https://www.ncbi.nlm.nih.gov/nuccore/MF773680>

Nodulisporium sp. (11_8)

<https://www.ncbi.nlm.nih.gov/nuccore/MF773681.1>
